# *Tetrastigma Hemsleyanum* Polysaccharide Suppresses Triple‐Negative Breast Cancer by Disrupting the Hippo‐YAP/TEAD4‐PDIA4 Axis and Endoplasmic Reticulum Stress Adaptation

**DOI:** 10.1002/advs.202519006

**Published:** 2026-04-02

**Authors:** Yini Shang, Wentao Si, Youxue Zhang, Jialin Liu, Huaixi Zhang, Yafei Li, Hongmeng Su, Wei Jiang, Zhishan Ding, Lihong Wang

**Affiliations:** ^1^ Fujian Key Laboratory of Tumor Immunotherapy the First Affiliated Hospital Fujian Medical University Fuzhou China; ^2^ National Regional Medical Center Binhai Campus of the First Affiliated Hospital Fujian Medical University Fuzhou China; ^3^ Department of Breast Surgery Harbin Medical University Cancer Hospital Harbin Heilongjiang China; ^4^ School of Medical Technology and Information Engineering Zhejiang Chinese Medical University Hangzhou China; ^5^ Department of Bioinformatics Fujian Key Laboratory of Medical Bioinformatics Institute of Precision Medicine School of Medical Technology and Engineering Fujian Medical University Fuzhou China

**Keywords:** endoplasmic reticulum stress, hippo‐YAP pathway, metastasis, *tetrastigma hemsleyanum* polysaccharide, triple negative breast cancer

## Abstract

Triple‐negative breast cancer (TNBC) exhibits addiction to chronic endoplasmic reticulum (ER) stress, which sustains an aggressive metastatic phenotype through activation of the unfolded protein response (UPR). Here, we identify a previously unrecognized “ER‐stress addiction” axis in which the Hippo pathway effector TEAD4 directly transcriptionally upregulates the ER chaperone PDIA4. We further demonstrate that this axis can be pharmacologically targeted by a natural polysaccharide. *Tetrastigma hemsleyanum* polysaccharide (THP) selectively activates the Hippo kinase cascade, leading to YAP phosphorylation, cytoplasmic sequestration, and subsequent degradation. This cascade attenuates YAP/TEAD4 interaction and abolishes TEAD4 DNA‐binding activity. Moreover, THP downregulates TEAD4 expression. These combined effects drive transcriptional suppression of PDIA4, catastrophic disruption of ER proteostasis, and ultimately lethal ER stress in TNBC cells. Functionally, THP inhibits migration, invasion, angiogenesis, and intracellular Ca^2^^+^ flux in vitro, and—importantly—blocks metastasis in patient‐derived organoids, zebrafish xenografts, and two syngeneic mouse models at non‐toxic doses. Multi‐omics analyses and rescue assays confirm the TEAD4‐PDIA4 axis as the core functional module. Our findings establish THP as a first‐in‐class, natural‐product‐based therapeutic that disrupts ER‐stress addiction in metastatic TNBC by targeting the Hippo‐YAP/TEAD4‐PDIA4 axis.

## Abstract 2

Triple‐negative breast cancer exploits chronic ER stress to drive metastasis. This study identifies a Hippo‐YAP/TEAD4‐PDIA4 axis as a critical mediator of ER‐stress addiction. The natural polysaccharide THP hierarchically disrupts this axis by activating Hippo signaling and downregulating TEAD4, suppressing PDIA4, and triggering lethal ER stress. THP exhibits potent anti‐metastatic efficacy across multiple preclinical models with a favorable safety profile, offering a first‐in‐class therapeutic strategy for metastatic TNBC.

## 1 Introduction

Triple‐negative breast cancer (TNBC) accounts for 15%–20% of all breast cancers yet is responsible for a disproportionate share of the ∼500 000 annual breast‐cancer deaths worldwide [1, 2]. Its aggressive metastatic behavior—driven by a complex network of signaling pathways enhancing cell migration, invasion, and therapy resistance—remains the major cause of treatment failure and poor survival outcomes [3]. Understanding the molecular mechanisms that sustain TNBC dissemination is therefore an urgent and unmet clinical need.

Natural products have long been a valuable source of anticancer therapeutics [4]. *Tetrastigma hemsleyanum*, a perennial vine traditionally used in Chinese medicine to treat fever, pneumonia, and viral meningitis [5, 6], produces over 150 secondary metabolites, including polyphenols and polysaccharides [7]. Recent studies have demonstrated potent anti‐inflammatory and antioxidant properties of its polysaccharide fraction—*Tetrastigma hemsleyanum* polysaccharides (THP) [8]; our preliminary data further indicate that THP inhibits TNBC proliferation in vitro [9]. This prompted us to investigate its potential role in suppressing metastasis.

A key driver of TNBC aggressiveness is the evolutionarily conserved Hippo pathway [10]. Activation of the MST1/2‐LATS1/2 kinase cascade leads to phosphorylation and cytoplasmic retention of YAP/TAZ; when inactive, YAP/TAZ translocates to the nucleus, binds TEAD transcription factors (particularly TEAD4), and activates pro‐metastatic gene expression [11, 12]. Nuclear YAP‐TEAD4 transcriptional activity promotes breast cancer progression and is associated with poor patient prognosis [13], yet its functional crosstalk with cellular stress response pathways remains poorly understood.

Among these stress‐adaptive systems is the endoplasmic reticulum (ER) unfolded protein response (UPR) [14]. Protein‐disulfide isomerase A4 (PDIA4), an ER‐resident chaperone and key UPR effector, is strongly upregulated in multiple cancers and correlated with poor survival [15, 16, 17]. Given that ER stress and UPR activation are negative prognostic factors in cancer [18], understanding the regulation and function of key effectors like PDIA4 becomes crucial. However, selective PDIA4 inhibitors are currently unavailable, and their transcriptional regulation is not well characterized.

This study demonstrates that THP disrupts ER‐stress addiction by targeting key nodes within this adaptive network. Specifically, THP activates the Hippo kinase cascade, which leads to the suppression of TEAD4‐mediated transcription of PDIA4. The consequent depletion of PDIA4 disrupts ER proteostasis, induces severe ultrastructural ER damage, and substantially impairs the migration, invasion, and metastatic capacity of TNBC cells both in vitro and in vivo. Through integrated models—including patient‐derived organoids, zebrafish xenografts, and murine systems—we validate the Hippo‐YAP/TEAD4‐PDIA4 axis as a therapeutically actionable vulnerability. Our findings establish THP as a first‐in‐class, natural‐product‐based agent that targets this axis, offering a translatable strategy for the treatment of metastatic TNBC.

## 2 Results

### 2.1 THP Inhibited the Metastasis of TNBC Cells

**FIGURE 1:**
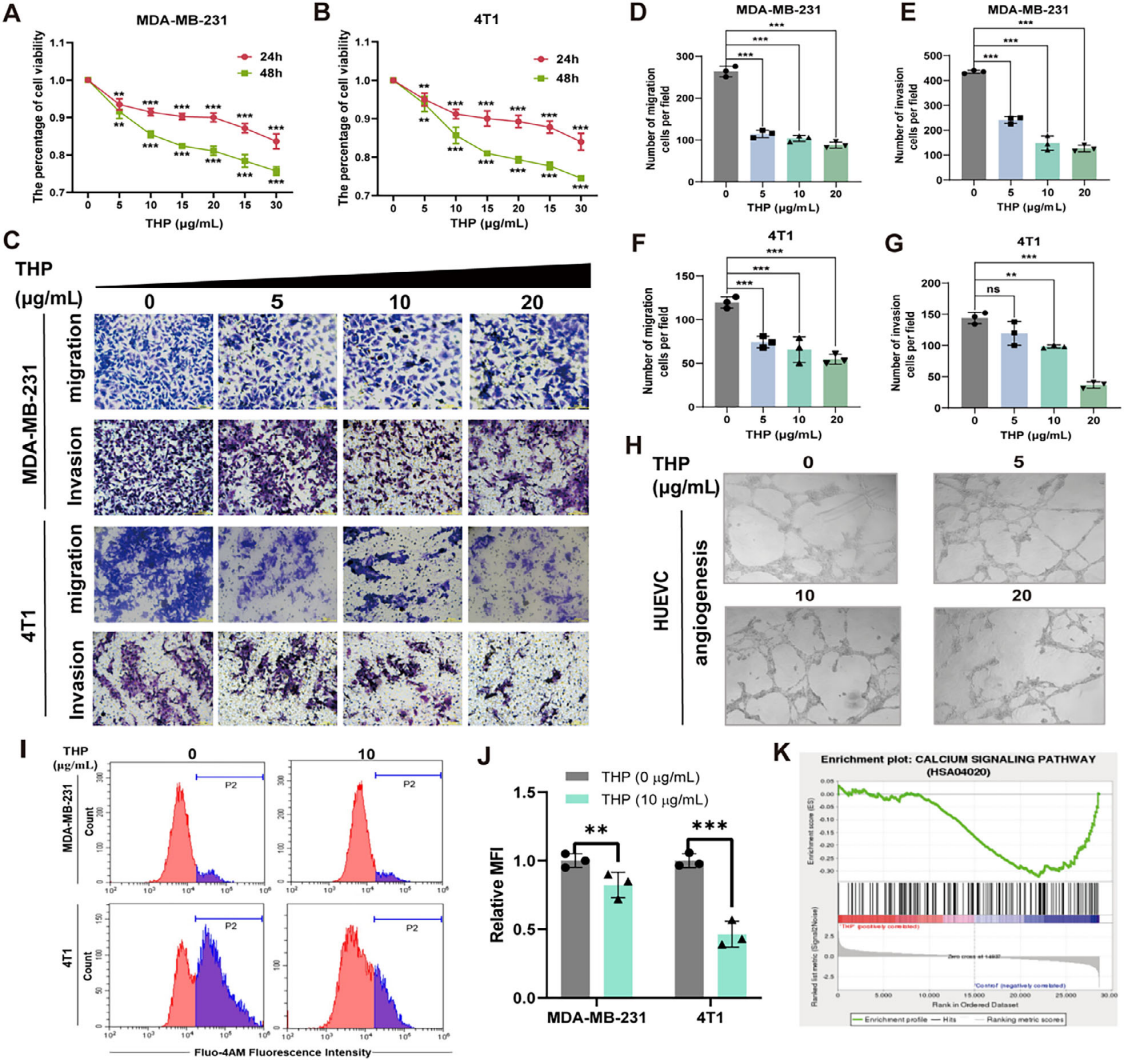
THP inhibited the metastasis of TNBC cells. (A,B) CCK‐8 assay showing the effects of THP on the viability of MDA‐MB‐231 and 4T1 cells after 24 and 48 h of treatment. (C) Transwell analysis of migration and invasion in MDA‐MB‐231 and 4T1 cells following treatment with varying concentrations of THP for 24 h. (D–G) Quantization results in (C). (H) Tube formation test of HUVEC cells treated with different concentrations of THP. (I) Representative flow cytometry histograms showing intracellular Ca^2^^+^ levels in MDA‐MB‐231 and 4T1 cells loaded with Fluo‐4 AM. Cells were treated without or with THP for 24 h. Fluorescence intensity was measured by flow cytometry. (J) Quantification of intracellular Ca^2^^+^ level in MDA‐MB‐231 and 4T1 cells treated without or with THP. Peak MFI values were normalized to the untreated group (mean set as 1.0) and are shown as fold change. (K) GSEA reveals the downregulation of Ca^2^^+^ signaling in MDA‐MB‐231 and 4T1 cells treated with THP (10 µg/mL) compared to the control group. Data represent mean ± SD. Significance in (A,B) and (D–G) was calculated using the one‐way ANOVA with Dunnett's multiple comparisons test. Statistical significance in (J) was determined by unpaired two‐tailed Student's *t*‐test. n ≥ 3; ^*^ *p* < 0.05; ^**^ *p* < 0.01; ^***^ *p* < 0.001.

After identifying the major components of the *Tetrastigma hemsleyanum* polysaccharide (THP), all in vivo and in vitro experiments in this study were conducted using a single batch (Batch No.: THP‐2023‐05‐Batch05) to ensure the chemical consistency of the experimental material. Systematic chemical characterization of this batch revealed a well‐defined composition and high purity. Specifically, the batch contained 83.5% total carbohydrate, 24.73% total uronic acid, and less than 0.01% protein (Figure S1 and Table S1). Monosaccharide composition analysis of THP was performed by high‐performance anion‐exchange chromatography with pulsed amperometric detection (HPAEC‐PAD) after TFA hydrolysis. The results revealed that THP is primarily composed of galactose (Gal), glucuronic acid (GlcA), and mannose (Man), with smaller amounts of arabinose (Ara), rhamnose (Rha), and glucose (Glc). The molar ratio of these monosaccharides was determined to be Gal: GlcA: Man: Ara: Rha: Glc = 35.77: 26.95: 20.90: 11.89: 2.95: 1.53, indicating that galactose is the predominant monosaccharide component (Figure S2 and Table S2). Furthermore, high‐performance liquid chromatography coupled with multi‐angle laser light scattering (HPLC‐MALLS) exhibited a symmetrical unimodal distribution, confirming good sample homogeneity (Figure S3). In addition, the fine structure of THP was characterized by nuclear magnetic resonance (NMR) spectroscopy (Figure S4). Together, these systematic characterization results establish a clear chemical fingerprint for this batch of THP, providing a reliable foundation for subsequent functional studies. Based on this, we further investigated the anti‐metastatic effects of THP against TNBC. We first evaluated the cytotoxic effects of THP on MDA‐MB‐231 and 4T1 cells using CCK‐8 assays over 24 and 48 h. At 24 h, THP (5–20 µg/mL) showed minimal cytotoxicity, with cell viability consistently above 90%. By contrast, prolonged exposure (48 h) inhibited proliferation in a concentration‐dependent manner, reducing viability to approximately 80% at 20 µg/mL (Figure 1A,B). We next examined the anti‐metastatic potential of THP using Transwell assays. Based on the 24 h cytotoxicity data, three concentrations (5, 10, and 20 µg/mL) were selected to avoid confounding effects from acute toxicity. THP significantly suppressed the migration and invasion of both TNBC cell lines in a dose‐dependent manner (Figure 1C–G). Notably, even at 5 µg/mL—a concentration with > 90% viability at 24 h—migration was reduced by approximately 50% in MDA‐MB‐231 cells and 40% in 4T1 cells. This dissociation between high cell viability and reduced metastatic capacity confirms that the anti‐invasive effects of THP are genuine and independent of reduced cell survival.

Recognizing tumor metastasis as a complex, multi‐step biological process, we employed an integrated experimental strategy to validate the anti‐metastatic efficacy of THP in TNBC models. We first evaluated the potential anti‐angiogenic property of THP, as angiogenesis is crucial for tumor metastasis. As shown in Figure 1H, treatment of HUVECs with THP (10 and 20 µg/mL) markedly suppressed their tube‐forming capacity, indicating a direct inhibitory effect on endothelial cell function. Calcium ions (Ca^2^^+^), which play an indispensable role in various physiological processes and the maintenance of organismal homeostasis, are also critically involved in regulating tumor metastasis. We therefore examined whether THP influences intracellular Ca^2^^+^ levels in TNBC cells. Using flow cytometry with the calcium indicator Fluo‐4 AM, we found that THP (10 µg/mL) treatment significantly reduced intracellular Ca^2^^+^ levels. As shown in Figure 1I,J, the relative mean fluorescence intensity (MFI) decreased to 0.82 ± 0.09 in MDA‐MB‐231 cells and to 0.46 ± 0.10 in 4T1 cells (expressed as fold change vs. untreated controls; *p* < 0.01). To assess cellular calcium storage status, a biochemical total calcium assay was performed. Quantification using a calcium detection kit across a range of drug concentrations confirmed that THP reduces total intracellular calcium content in a dose‐dependent manner (Figure S5A,B). Consistent with these findings, transcriptomic analysis indicated a broad attenuation of calcium‐related signaling upon THP treatment. This conclusion was further supported by Gene Set Enrichment Analysis (GSEA) of RNA‐seq data, which revealed significant suppression of calcium‐associated pathways (Figure 1K). Collectively, these results demonstrate that THP exerts notable anti‐metastatic effects on TNBC cells, manifested through a broad spectrum of phenotypic changes and marked efficacy.

### 2.2 THP Significantly Regulates the Hippo Pathway

**FIGURE 2:**
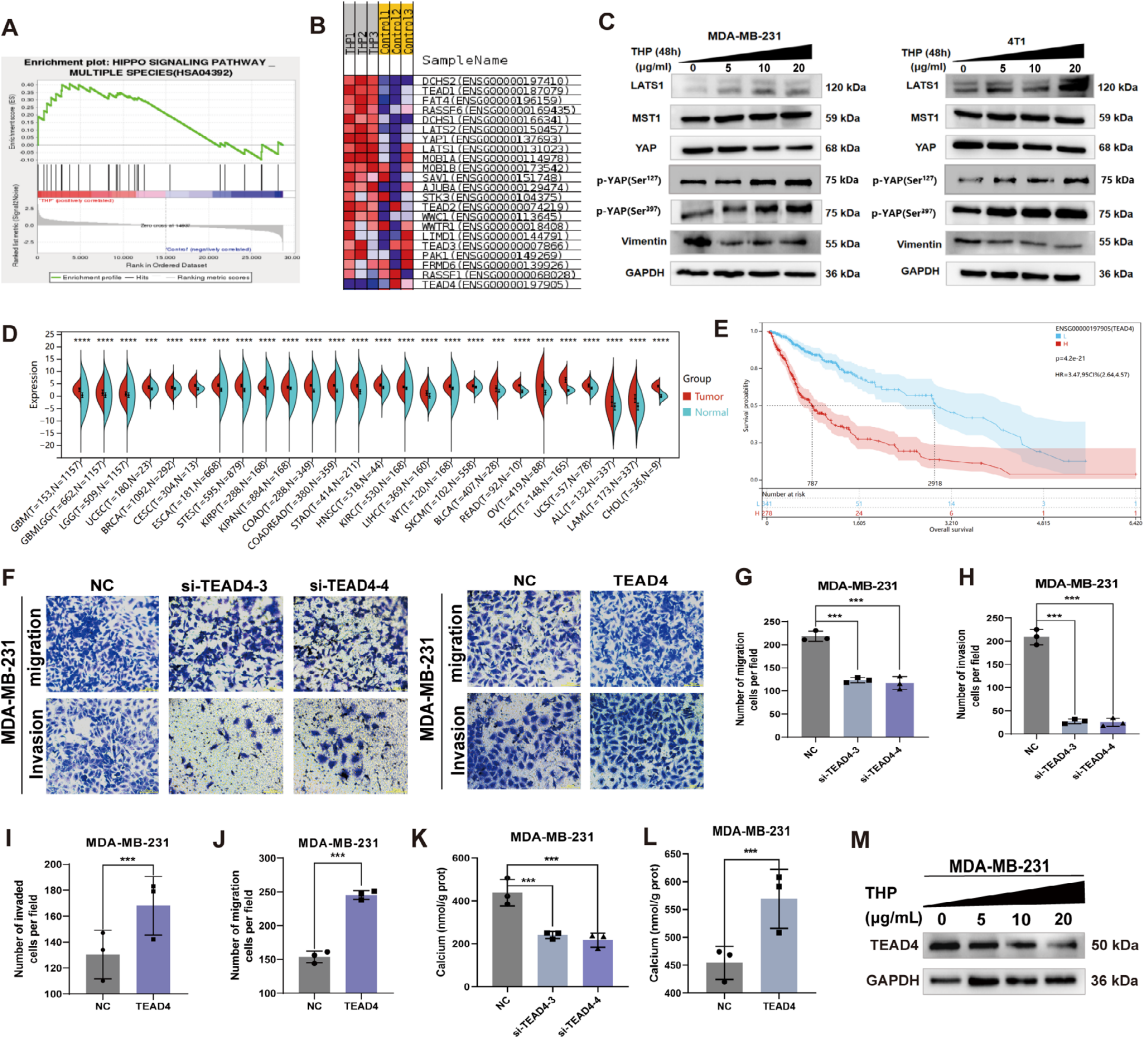
TEAD4 is an important target for TNBC metastasis. (A) GSEA revealed upregulation of Hippo signaling in the THP‐treated (10 µg/mL) group compared to the control. (B) The heat map shows the DEGs in MDA‐MB‐231 cells compared to the control after 24 h of THP (10 µg/mL) treatment. (C) Western blot analysis of MST1, LATS1, YAP, p‐YAP, and Vimentin after treatment of THP for 48 h in MDA‐MB‐231 and 4T1 cells at different concentrations of THP (5, 10, 20 µg/mL), respectively. (D) Expression of TEAD4 in pancarcinoma. (E) Impact of TEAD4 expression on survival of TNBC. (F) Transwell assays demonstrating the influence of TEAD4 overexpression and knockdown on the migration and invasion capacities of MDA‐MB‐231 cells. (G–J) Quantitative results of (F). (K,L) Total cellular Ca^2^^+^ levels were measured in MDA‐MB‐231 cells following TEAD4 overexpression or knockdown. (M) Western blot analysis of TEAD4 expression in MDA‐MB‐231 cells treated with THP at concentrations of 5, 10, and 20 µg/mL for 48 h. Data represent mean ± SD. Significance in (G,H) and (K) was calculated using the one‐way ANOVA with Dunnett's multiple comparisons test. Significance in (I,J) and (L) was calculated using the Unpaired T‐test. n = 3; ^*^ *p* < 0.05; ^**^ *p* < 0.01; ^***^ *p* < 0.001. [Correction added on 7 April 2026 after first online publication: Figure 2 is updated.]

Building upon RNA‐seq results, we further investigated the mechanism by which THP inhibits TNBC metastasis. GSEA revealed an upregulation of Hippo signaling in the THP‐treated group compared to the control (Figure 2A). Subsequently, analysis of the RNA‐seq data identified differentially expressed genes (DEGs) associated with the Hippo pathway between the THP and control groups (Figure 2B). To validate the RNA ‐seq results, we performed western blot analysis to examine key proteins within the Hippo signaling pathway. The results showed that THP treatment significantly upregulates the expression of the core Hippo kinases MST1 and LATS1. This activation of the kinase cascade was accompanied by characteristic suppression of YAP, as indicated by reduced total YAP protein levels and enhanced phosphorylation at Ser127 and Ser397. These findings indicate that THP sequesters YAP in the cytoplasm through phosphorylation at Ser127 and promotes YAP degradation via phosphorylation at Ser397, thereby limiting its nuclear translocation and subsequent functional activity. Additionally, the expression of the metastasis‐related protein Vimentin decreased in a dose‐dependent manner after THP treatment (Figure 2C; Figure S6). These results indicate THP‐induced cytoplasmic sequestration and degradation and functional inactivation of YAP, thereby effectively inhibiting its nuclear translocation and preventing the formation of functional transcriptional complexes with TEAD family members. Notably, TEAD4, a key downstream transcription factor of the Hippo pathway, has been identified as a critically important effector in tumor progression. Comprehensive analysis of clinical datasets revealed consistent overexpression of TEAD4 across multiple cancer types (Figure 2D). More importantly, survival analysis demonstrated that high TEAD4 expression is significantly associated with poorer prognosis in TNBC patients (log‐rank *p* < 0.001, hazard ratio = 3.47, 95% CI: 2.54–4.57), underscoring its pivotal role in TNBC pathogenesis (Figure 2E).

To further investigate the role of TEAD4 in TNBC metastasis and verify its potential as a key target of THP, we designed and synthesized four TEAD4‐specific small interfering RNAs (siRNAs) with low off‐target risk, as well as mammalian expression plasmids for TEAD4 overexpression. RT‐qPCR (Figure S7A,B) and western blot (Figure S7C,D) analyses demonstrated that si‐TEAD4‐3, si‐TEAD4‐4, and the overexpression group elicited significant effects. Based on these results, si‐TEAD4‐3 and si‐TEAD4‐4 were selected for further phenotypic studies. Initial RT‐qPCR expression profiling revealed that MDA‐MB‐231 cells exhibit significantly higher TEAD4 mRNA levels compared to other breast cancer cell lines (Figure S7E), establishing this cell line for subsequent mechanistic studies. We found that genetic modulation of TEAD4 had distinct effects on cellular functions. While genetic knockdown or overexpression of TEAD4 did not significantly affect proliferation (Figure S7F,G), both interventions profoundly altered metastatic potential, with TEAD4 knockdown significantly attenuating and its overexpression markedly enhancing cell migration and invasion capabilities (Figure 2F–J). These findings demonstrate that, under basal conditions, TEAD4 primarily regulates metastatic capacity rather than cell proliferation. Concomitantly, total Ca^2^^+^ levels mirrored these changes. TEAD4 overexpression elevated Ca^2^^+^ levels, supplying the driving force for metastasis, while TEAD4 silencing reduced Ca^2^^+^ levels (Figure 2K,L). Moreover, TEAD4 expression declined in a concentration‐dependent manner following THP treatment (Figure 2M). These findings establish TEAD4 as a pivotal driver of TNBC metastasis and progression, while underscoring the therapeutic potential of THP in TNBC through its regulation of the Hippo pathway, primarily by targeting TEAD4.

### 2.3 THP Suppresses PDIA4 Expression and Induces ER Stress by Modulating TEAD4 Transcriptional Activity

**FIGURE 3:**
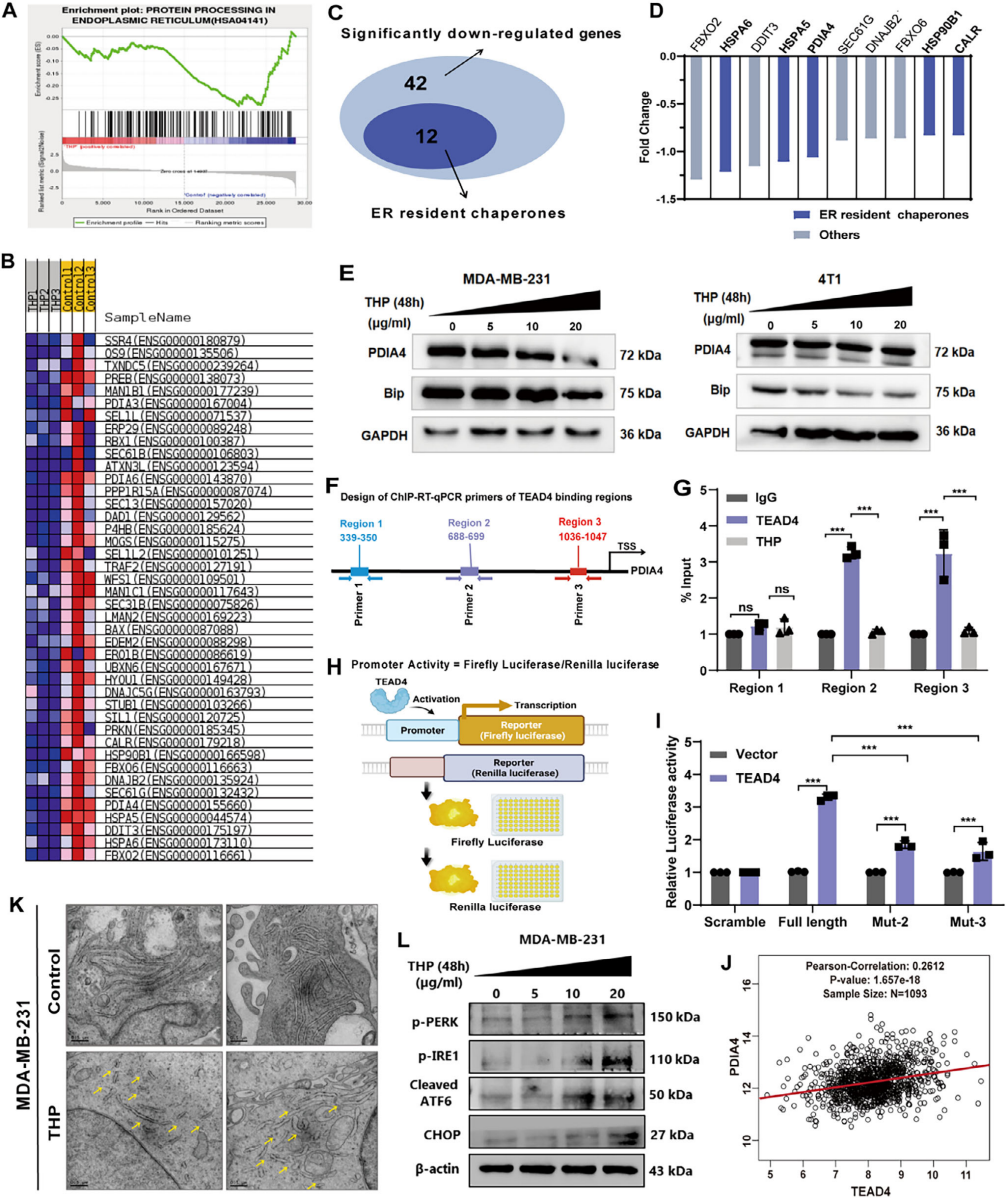
THP inhibits TNBC cells' protein processing and regulates ERS. (A) GSEA revealed significant downregulation of ER protein processing pathways in the THP ‐treated group compared to the control. (B) The heatmap illustrates DEGs associated with THP treatment in MDA‐MB‐231 cells relative to the control group following a 24 h exposure. (C) The Venn diagram illustrates the protein classification corresponding to the DEGs. (D) The top ten genes most significantly down‐regulated following THP treatment. (E) Western blot analysis of PDIA4 and Bip expression in MDA‐MB‐231 and 4T1 cells treated with varying concentrations of THP (5, 10, and 20 µg/mL) for 48 h. (F) Diagram showing the locations of the three ChIP primer design sites. (G) The ChIP assay demonstrated the interaction between TEAD4 and PDIA4 both prior to and following THP treatment. (H) Procedure for dual‐luciferase reporter assays. (I) The dual‐luciferase reporter assay confirmed that TEAD4 acts as a positive regulator of PDIA4. (J) Analysis of the TCGA database revealed a correlation between PDIA4 and TEAD4 expression in 1093 clinical samples. (K) Electron micrographs of MDA‐MB‐231 cells, untreated (control) or treated with THP (10 µg/mL) for 24 h. Scale bar: 0.5 µm. (L) Western blot analysis of p‐PERK, p‐IRE1, cleaved ATF6, and CHOP expression in MDA‐MB‐231 cells treated with varying concentrations of THP (5, 10, and 20 µg/mL) for 48 h. Data represent mean ± SD Significance in (G,I) was calculated using the one‐way ANOVA with Tukey's multiple comparisons test.  = 3; ^***^ *p* < 0.001; ns, not significant.

The GSEA results further indicated enrichment of ER protein processing pathways in the THP‐treated group compared to the control (Figure 3A). A total of 42 genes were implicated in this process, and a subset of representative genes significantly regulated by THP within this pathway is displayed in Figure 3B. Among the encoded proteins, ER chaperones are critical for proteostasis, and their dysfunction can have widespread consequences. We classified the 42 DEGs and identified 12 transcripts encoding chaperone proteins (Figure 3C). When ranking the top ten most significantly downregulated genes by fold‐change, five were identified as ER chaperones, including HSPA5 (which encodes Bip) and PDIA4 (Figure 3D), suggesting that THP may disrupt ER function through modulation of multiple chaperones. To prioritize a target for functional investigation, we first validated the protein expression of the two prominent candidates, Bip and PDIA4. Western blot analysis demonstrated that THP treatment reduced both PDIA4 and Bip protein levels in MDA‐MB‐231 and 4T1 cells (Figure 3E). To determine whether PDIA4 or HSPA5 serves as a direct transcriptional target of the YAP/TEAD4 axis, we first predicted potential TEAD4 binding sites within their promoter regions using the JASPAR Core database. Subsequent chromatin immunoprecipitation quantitative PCR (ChIP‐qPCR) assays revealed that TEAD4 does not significantly bind to any of the three predicted regions in the HSPA5 promoter in MDA‐MB‐231 cells (Figure S8A). In stark contrast, TEAD4 directly and specifically binds to two of the three predicted regions (sites 2 and 3) within the PDIA4 promoter. Notably, THP treatment markedly reduced TEAD4 occupancy at these binding sites (Figure 3F,G). This attenuated enrichment may be linked to the decreased total TEAD4 protein levels induced by THP, indicating that THP effectively disrupts the TEAD4‐PDIA4 regulatory axis. Furthermore, dual‐luciferase reporter assays confirmed that TEAD4 positively regulates PDIA4 transcription, with both promoter regions 2 and 3 contributing to this activation (Figure 3H,I). These findings are consistent with TCGA database predictions, which indicate a significant positive correlation between the expression levels of TEAD4 and PDIA4 (Figure 3J). Based on these findings, PDIA4 was selected for further functional and mechanistic investigation.

We next sought to characterize the functional consequences of PDIA4 downregulation and broader ER pathway disruption. Transmission electron microscopy revealed severe ultrastructural abnormalities in THP‐treated cells, including ER fragmentation and swelling, indicative of profound ER dysfunction, unlike the normal ER morphology seen in controls (Figure 3K). At the molecular level, western blot analysis revealed that THP treatment significantly upregulated key UPR sensors (PERK, IRE1α, cleaved ATF6) and the pro‐apoptotic effector CHOP in a dose‐dependent manner (Figure 3L). These data collectively indicate that the ER stress response triggered by THP is dominated by a strong and sustained pro‐apoptotic output (via the PERK‐CHOP axis), which overwhelms cellular adaptive capacity. To benchmark this response, we treated cells with tunicamycin, a canonical N‐glycosylation inhibitor and ER stress inducer. As expected, tunicamycin robustly upregulated the expression of classic adaptive UPR genes, HSPA5 and PDIA4 (Figure S8B–D). This canonical upregulation starkly contrasted with the transcriptional downregulation of these same chaperones by THP, suggesting THP provokes a distinct, maladaptive form of ER stress that contributes to its cytotoxic effects.

### 2.4 PDIA4 Drives TNBC Metastasis and is Transcriptionally Regulated by TEAD4

**FIGURE 4:**
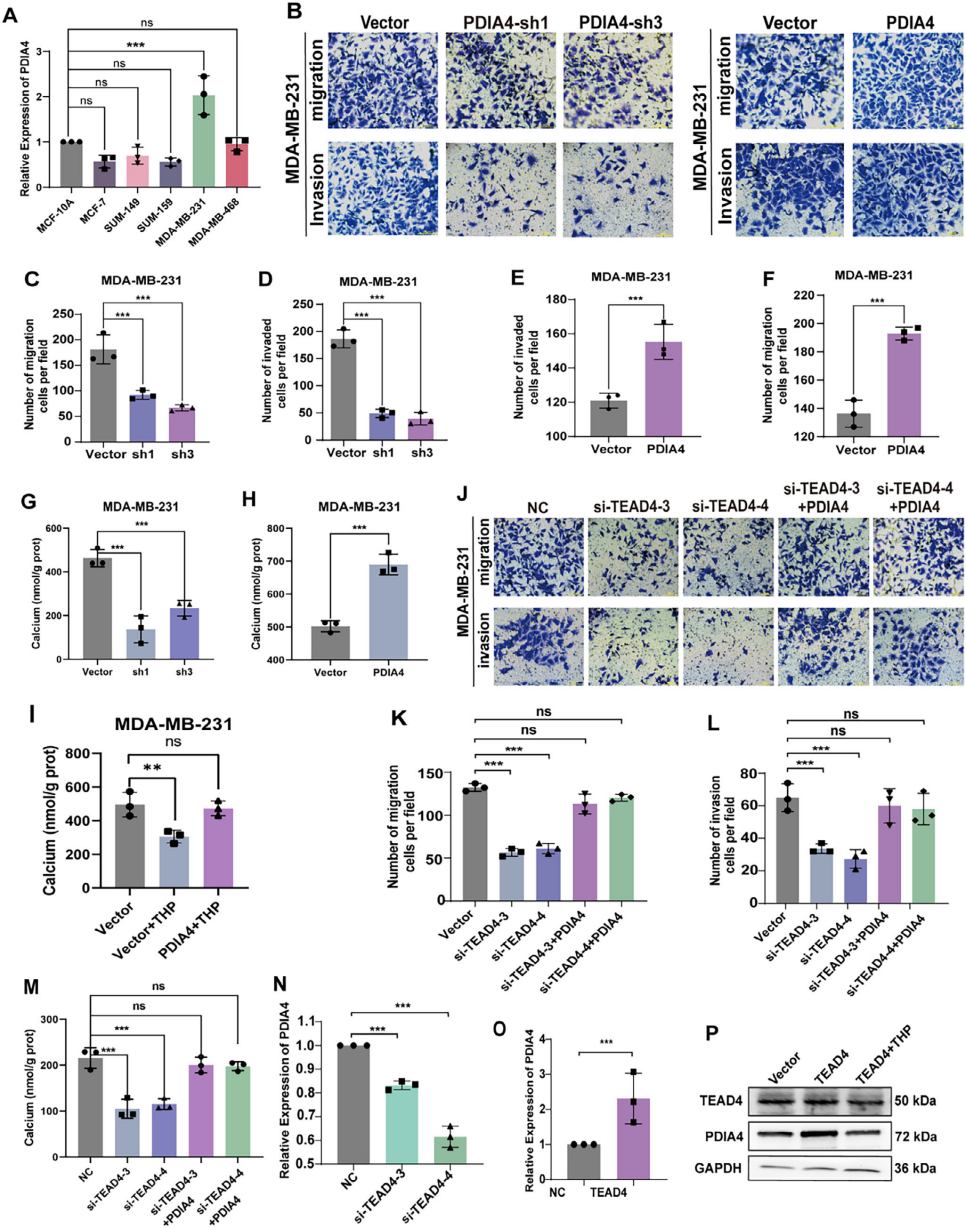
PDIA4 is a functional downstream effector of TEAD4 in TNBC metastasis. (A) RT‐qPCR quantified the relative mRNA expressions of PDIA4 in normal mammary epithelial cell lines MCF‐10A and breast cancer cell lines. (B) Transwell migration and invasion assays in MDA‐MB‐231 cells confirmed alterations in cellular migratory and invasive capacities following PDIA4 overexpression and knockdown. (C–F) Quantitative analysis of (B). (G,H) Total cellular Ca^2^^+^ levels were measured in MDA‐MB‐231 cells following 24 h of PDIA4 knockdown or overexpression. (I) Total cellular Ca^2^^+^ levels were measured in MDA‐MB‐231 cells following 24 h of THP treatment and PDIA4 overexpression. (J) Transwell assay detected the migration and invasion ability of MDA‐MB‐231 cells following TEAD4 knockdown alone or in combination with PDIA4 overexpression. (K,L) Quantitative analysis of data presented in panel (J). (M) Total cellular Ca^2^^+^ levels were measured in MDA‐MB‐231 cells 24 h after TEAD4 knockdown alone or in combination with PDIA4 overexpression. (N,O) The expression of PDIA4 mRNA in MDA‐MB‐231 cells was detected after TEAD4 knockdown and overexpression. (P) The protein expression of TEAD4 and PDIA4 was detected by western blot after TEAD4 overexpression and THP treatment. Data represent mean ± SD, Significance in (A), (C,D), (G,I), and (K–N) was calculated using the one‐way ANOVA with Dunnett's multiple comparisons test. Significance in (E,F), (H), and (O) was calculated using the Unpaired T‐test. n = 3; ^*^ *p* < 0.05; ^**^ *p* < 0.01; ^***^ *p* < 0.001; ns, not significant.

To investigate the function of PDIA4 in TNBC metastasis, we first assessed its expression pattern. PDIA4 mRNA was significantly upregulated in the TNBC cell line MDA‐MB‐231 compared to the normal breast epithelial cell line MCF‐10A and other breast cancer cells (Figure 4A). We therefore selected MDA‐MB‐231 for subsequent functional studies. To define PDIA4's role, we established knockdown and overexpression models using two independent shRNAs (sh1 and sh3) and a PDIA4 expression construct. Successful modulation of PDIA4 at the mRNA and protein levels was confirmed by RT‐qPCR and western blot, respectively (Figure S9A–D). Functionally, PDIA4 knockdown significantly inhibited cell migration and invasion, while its overexpression markedly enhanced these malignant behaviors (Figure 4B–F). We next examined whether PDIA4 regulates calcium signaling. Knockdown of PDIA4 reduced total cellular Ca^2^^+^ levels, whereas its overexpression led to elevated Ca^2^^+^ concentrations (Figure 4G,H), implicating that PDIA4 plays a role in regulating cellular calcium levels. In parallel, we observed that THP treatment downregulated PDIA4 expression and suppressed metastasis, accompanied by a decrease in intracellular Ca^2^^+^ levels. To determine whether this Ca^2^^+^ reduction is a direct consequence of PDIA4 loss rather than a nonspecific outcome of ER stress or cell death, we performed a rescue experiment by re‐expressing PDIA4 in THP‐treated cells. Restoration of PDIA4 significantly reversed the THP‐induced decline in Ca^2^^+^ levels (Figure 4I), providing direct evidence that PDIA4 downregulation is causally linked to altered Ca^2^^+^ homeostasis in this context. These results establish PDIA4 as a critical promoter of TNBC progression and a potential therapeutic target.

We next sought to explore the mechanism of THP by defining the regulatory relationship between TEAD4 and PDIA4. Silencing TEAD4 markedly attenuated cell migration and invasion, and these phenotypes were fully rescued by re‐expressing PDIA4 (Figure 4J–L). Accordingly, TEAD4 depletion lowered total cellular Ca^2^^+^ levels, and PDIA4 restoration reversed this deficit (Figure 4M). These loss‐of‐function and rescue experiments position PDIA4 as an essential functional effector downstream of TEAD4. Consistent with this, RT‐qPCR analysis revealed that PDIA4 mRNA expression decreased following TEAD4 knockdown, while overexpression of TEAD4 led to its upregulation (Figure 4N,O). This regulatory link was confirmed at the protein level, where TEAD4 overexpression upregulated PDIA4, and this induction was effectively suppressed by THP treatment (Figure 4P). In summary, our results define the TEAD4‐PDIA4 axis as a critical downstream pathway mediating the anticancer efficacy of THP.

### 2.5 THP Inhibits TNBC Metastasis in a Syngeneic Mouse Model

**FIGURE 5:**
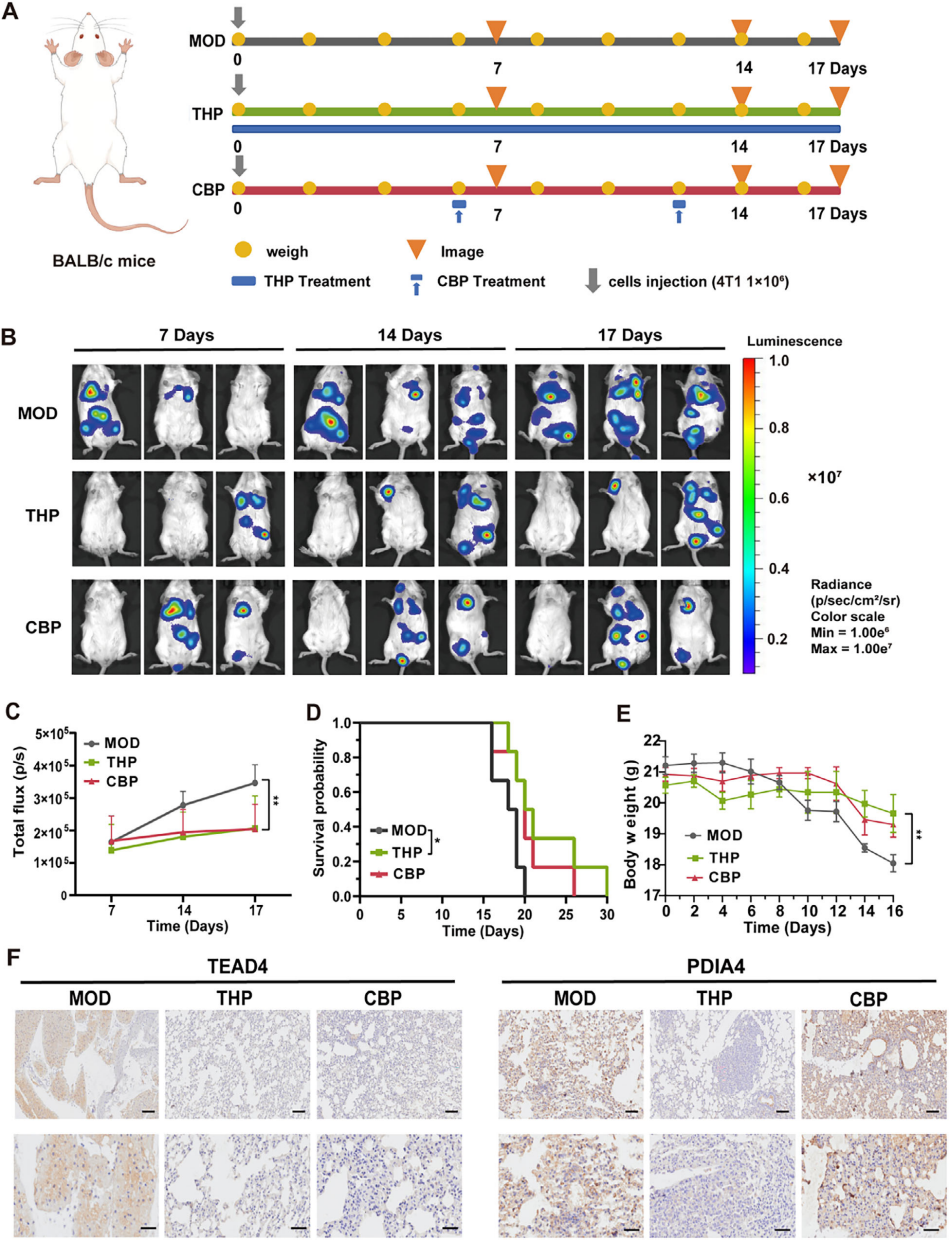
THP inhibits TNBC metastasis in vivo. (A) Schematic diagram of the experimental design in a mouse model inoculated with 4T1‐luc cells. (B) Representative in vivo bioluminescence images showing tumor metastasis. (C) Quantification of mean tumor bioluminescence flux over time under the indicated conditions. (D) Kaplan–Meier survival analysis showed the prognosis of each mice group. (E) Body weights were monitored over time across all experimental groups. (F) Representative images and quantification of IHC staining for TEAD4 and PDIA4 in lung tissue sections containing metastatic lesions. Data are presented as mean ± SD. Statistical significance in (C,E) was determined by one‐way ANOVA with Dunnett's multiple comparisons test. n = 6 mice per group; ^*^ *p* < 0.05; ^**^ *p* < 0.01; ns, not significant.

Then we evaluated the anti‐metastatic efficacy of THP against TNBC in vivo. All BALB/c mice were inoculated with luciferase‐expressing 4T1 cells via tail vein injection to establish tumor metastasis models. The mice were divided into three groups: the model control (MOD) group received daily oral gavage of normal saline; the THP group was administered THP (20 mg/kg) daily via oral gavage; and a positive control group received intraperitoneal injections of carboplatin (CBP; 50 mg/kg) once every 6 days (Figure 5A). In vivo tumors were imaged using the IVIS system at 7, 14, and 17 days after the 4T1 cells was implanted (Figure 5B). The total flux intensity served as an indicator of tumor size in each mouse. Periodic flux monitoring revealed that both the THP and CBP groups showed significantly lower total flux compared to the MOD group, suggesting comparable therapeutic efficacy between the THP and CBP treatments (Figure 5C). Compared to the MOD group, mice in the THP group exhibited prolonged survival and a slower rate of body weight loss (Figure 5D,E). Importantly, THP treatment did not induce detectable toxicity in major organs, as corroborated by histopathological analysis showing no apparent lesions in H&E‐stained sections of the liver, kidney, heart, or spleen (Figure S10). Representative immunohistochemical (IHC) images of TEAD4 and PDIA4 revealed pronounced inter‐group differences in tumor progression. In the MOD group, strong expression of both TEAD4 and PDIA4 highlighted their contribution to TNBC development. THP treatment markedly reduced the expression of both proteins. In contrast, CBP significantly decreased TEAD4 levels but only marginally suppressed PDIA4 (Figure 5F). These results suggest that TEAD4 and PDIA4 represent critical therapeutic targets of THP, whereas CBP appears ineffective in robustly inhibiting PDIA4.

### 2.6 THP Inhibits TNBC in Multiple Preclinical Models

**FIGURE 6:**
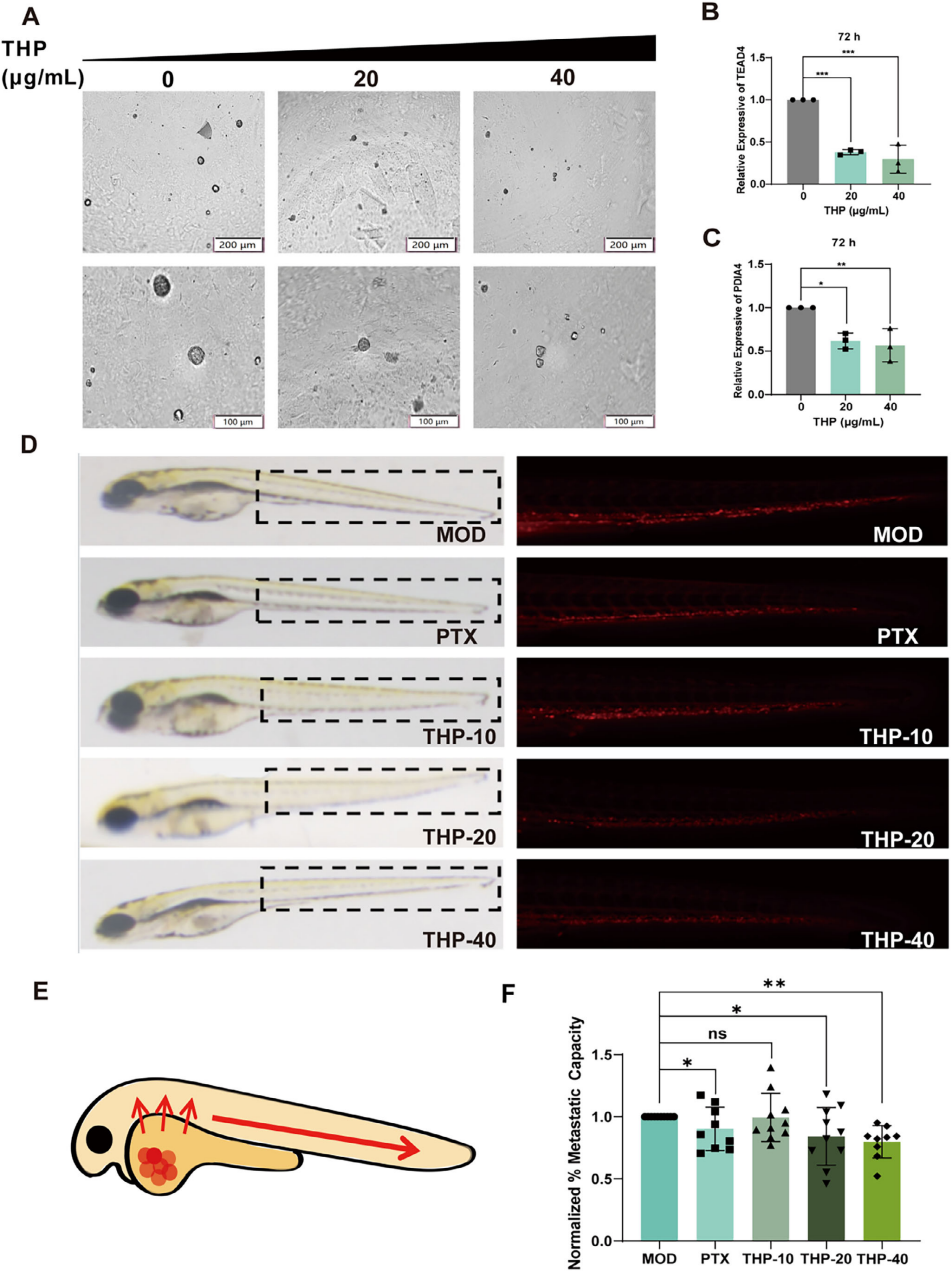
THP inhibits TNBC in multiple preclinical models. (A) Morphological comparison of TNBC organoids between the control group and THP‐treated groups at different concentrations. (B,C) RT‐qPCR analysis of relative mRNA expression levels of TEAD4 and PDIA4 in TNBC organoids (n = 3 independent organoid samples, each measured in triplicate). (D) The zebrafish model was employed to validate the inhibitory effect of THP on the migration of MDA‐MB‐231 cells. (E) Schematic diagram of the zebrafish xenograft model established using MDA‐MB‐231 cells. (F) Quantification of MDA‐MB‐231 cell migration distance in the zebrafish model from panel (D) (n = 10 zebrafish per group; experiment repeated independently three times). Data represent mean ± SD, significance in (B,C,F) was calculated using the one‐way ANOVA with Dunnett's multiple comparisons test. ^*^ *p* < 0.05; ^**^ *p* < 0.01; ***p < 0.001; ns, not significant.

Subsequently, we established multiple experimental models to further validate the efficacy of THP. As illustrated in Figure 6A, TNBC organoids were treated with two different concentrations of THP (20 and 40 µg/mL). Microscopic examination revealed a dose‐dependent reduction in both the diameter and number of organoids. Concurrently, mRNA was extracted from the organoids, and RT‒qPCR analysis demonstrated that the expression levels of TEAD4 and PDIA4 were also decreased in a dose‐dependent manner (Figure 6B,C). To intuitively evaluate the anti‐metastatic efficacy of THP against TNBC, fluorescence‐labeled MDA‐MB‐231 cells were injected into zebrafish, and metastasis was quantified based on the dissemination distance toward the tail region. Paclitaxel (PTX, 1 µg/mL) was used as a positive control, while THP was tested at three concentrations (10, 20, and 40 µg/mL) (Figure 6D). A schematic diagram illustrating the experimental workflow of the zebrafish study is provided in Figure 6E. The results demonstrated that medium‐dose THP (20 µg/mL) exhibited efficacy comparable to PTX, whereas high‐dose THP (40 µg/mL) resulted in superior inhibition of metastasis compared to the PTX group (Figure 6F). These findings indicate that THP exerts potent anti‐TNBC effects both in vitro and in vivo across multiple models, demonstrating consistent and robust efficacy.

### 2.7 Elevated PDIA4 Expression Accelerates In Vivo Metastasis

**FIGURE 7:**
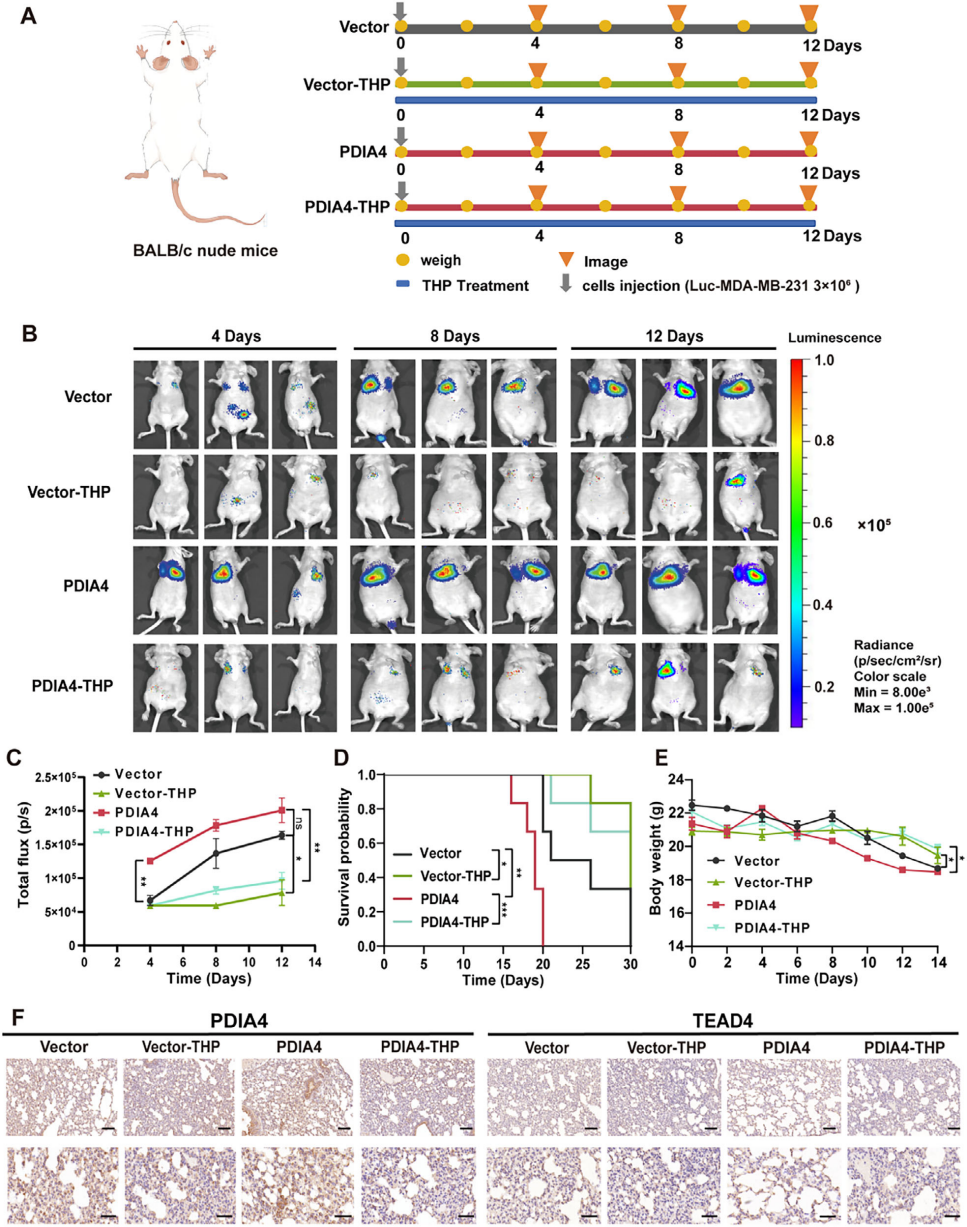
Elevated PDIA4 expression enhances the metastatic potential of TNBC. (A) Schematic illustration of the experimental design. (B) Representative in vivo bioluminescence imaging of tumor growth and metastasis in nude mice. (C) Quantification of mean tumor bioluminescence flux over time under the indicated conditions. (D) Kaplan–Meier survival analysis showed the prognosis of each mice group. (E) Body weight changes in different experimental groups. (F) Representative images of IHC staining for PDIA4 and TEAD4 in lung tissue sections from each group. Data are presented as mean ± SD. Statistical significance in (C,E), and the quantitative analysis in (F) was determined by one‐way ANOVA with Dunnett's multiple comparisons test. n = 6 mice per group; ^*^ *p* < 0.05; ^**^ *p* < 0.01; ^***^ *p* < 0.001; ns, not significant.

To further elucidate the role of PDIA4 in TNBC progression, we designed an in vivo experiment using four groups of nude mice. Two groups received tail‐vein injection of luciferase‐tagged MDA‐MB‐231 cells transduced with LV‐PDIA4, whereas the other two groups were injected with an equivalent number of LV‐Control MDA‐MB‐231 cells. Within each group, one subgroup received the vehicle alone as a control, whereas the other subgroup was administered THP (Figure 7A). Representative bioluminescence images at 4, 8, and 12 days visualized the dynamic growth and metastatic processes of tumors in each group (Figure 7B). Quantitative analysis revealed that PDIA4 overexpression significantly accelerated the onset of tumor metastasis compared to the Vector control group. Notably, THP administration effectively inhibited TNBC metastasis in both the PDIA4 overexpression and Vector control groups (Figure 7C). Survival analysis and body weight monitoring revealed that mice in the PDIA4 overexpression group exhibited the shortest survival time, the most rapid cancer progression, and a pronounced decrease in body weight. In contrast, THP treatment significantly extended survival and alleviated weight loss in these nude mice (Figure 7D,E). IHC staining of PDIA4 and TEAD4 in mouse lung tissues further corroborated our findings. As shown in Figure 7F, PDIA4 overexpression led to a pronounced increase in its expression in lung tissue, an effect that was potently suppressed by THP treatment. Concurrently, THP treatment led to a substantial downregulation of TEAD4 expression in both the Vector and PDIA4 overexpression groups. The coordinated downregulation of TEAD4 and PDIA4 following THP administration suggests that these proteins act as key targets mediating the anti‐tumor effects of THP.

Having experimentally established PDIA4 as a key target of THP, we next explored its broader clinical significance through bioinformatics analyses. As a chaperone protein, PDIA4 facilitates the abundant secretion of proteins and exacerbates ERS when overexpressed. It is broadly up‐regulated across diverse malignancies (Figure S11A), with TNBC displaying the highest levels of any subtype (Figure S11B). Importantly, high PDIA4 expression predicts significantly worse clinical outcomes (Figure S11C). Comprehensive analysis of large clinical cohorts revealed a positive correlation between PDIA4 expression and tumor mutation burden (TMB) across multiple cancer types, including breast cancer. These data position PDIA4 as a potential surrogate indicator of mutational load (Figure S11D). Furthermore, tissue‐based analyses revealed that PDIA4 levels are markedly elevated across multiple TNBC subtypes and in tumor‐adjacent tissues relative to normal breast tissue (Figure S11E), underscoring its pivotal role in breast cancer progression and therapeutic response. As shown in Figure S11F, the overall survival (OS) of patients with breast invasive carcinoma from The Cancer Genome Atlas Breast Invasive Carcinoma (TCGA‐BRCA) cohort, stratified by single‐sample gene‐set enrichment analysis (ssGSEA) scores. The Kaplan–Meier analysis revealed a significant survival difference between the two groups: patients in the high protein‐secretion group had a significantly higher risk of mortality than those in the low protein‐secretion group. Figure S11G displays the ssGSEA‐derived UPR scores within the TCGA‐BRCA cohort. Kaplan–Meier analysis revealed a marked survival difference between the low‐ and high‐protein‐secretion groups: patients with elevated UPR scores exhibited a significantly poorer prognosis and a markedly higher risk of mortality compared with those in the low‐score group. These findings demonstrate that the maintenance and repair mechanisms underlying tumor progression are driven by heightened protein secretion and a pronounced UPR, processes intimately linked to ERS. The endoplasmic reticulum chaperone PDIA4 plays a central role in this context. Thus, targeting PDIA4 expression to attenuate protein secretion and disrupt the ERS‐mediated repair response may represent a promising therapeutic strategy for suppressing tumor progression.

### 2.8 PDIA4 Modulates In Vivo Metabolic Homeostasis

**FIGURE 8:**
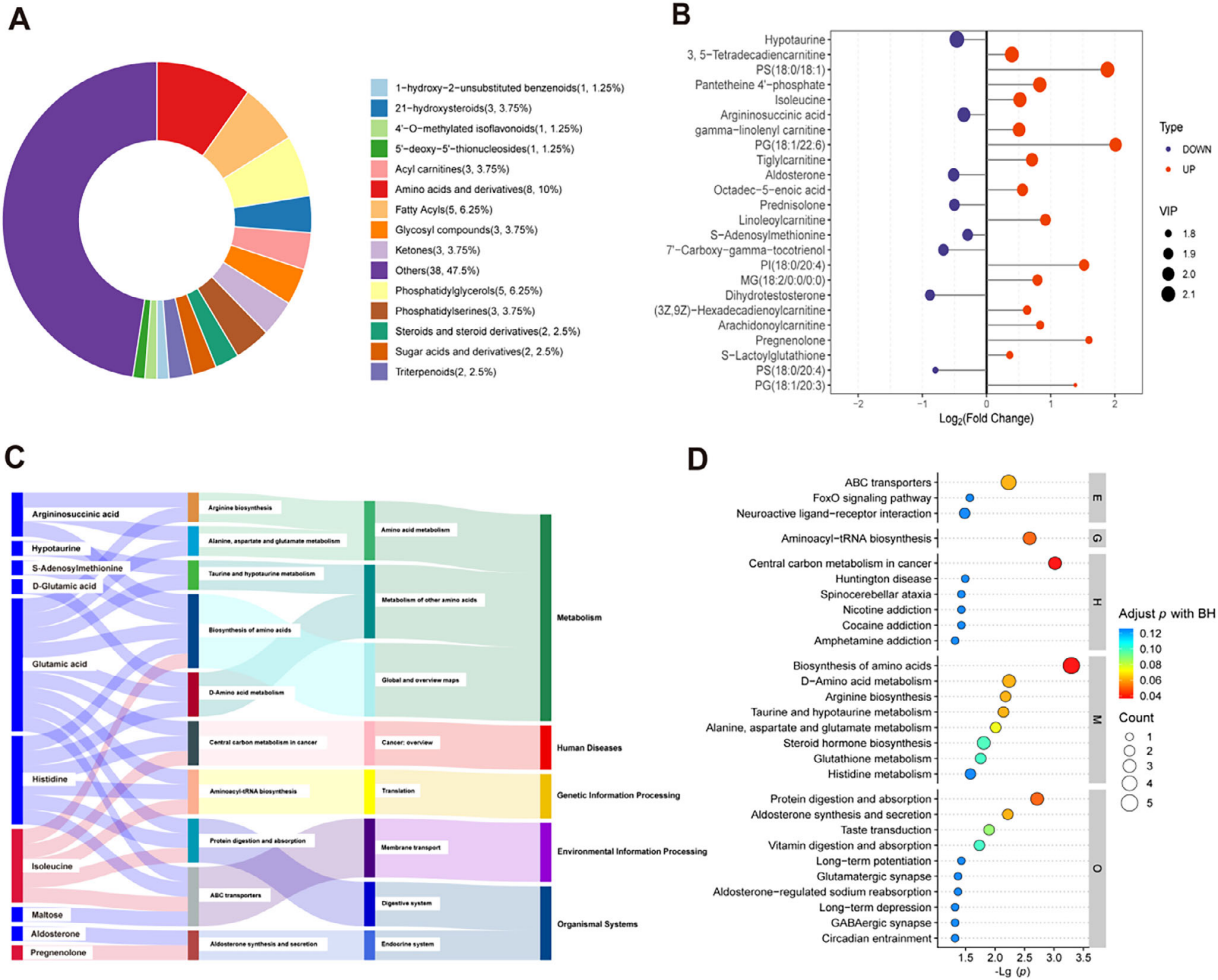
The in vivo metabolism is disturbed by the overexpression of PDIA4. (A) Loop diagram showing the number and proportion of differential metabolites between PDIA4‐overexpressing and Vector control groups. (B) Comparison of the top 24 VIP scores between PDIA4 and Vector groups. Point size: VIP score value. Red: Upregulated features. Blue: Downregulated features. (C) Hierarchical clustering of differential metabolites between PDIA4 and Vector groups (top 50 VIP features). (D) Bubble plot showing the top 30 enriched KEGG pathways for differential metabolites between PDIA4 and Vector groups.

To systematically delineate how PDIA4 reshapes in vivo metabolism and influences metastatic progression, we performed untargeted metabolomics profiling of lung metastatic lesions from tumor‐bearing mice. PDIA4 overexpression elicited profound metabolic reprogramming, with the most pronounced perturbations converging on amino‐acid and fatty‐acid pathways (Figure 8A). Variable Importance in Projection (VIP) analysis identified 24 signature metabolites that collectively define the metabolic reprogramming state induced by PDIA4 overexpression. Metabolomic profiling revealed that PDIA4 drives a metabolic configuration conducive to metastatic dissemination. Increased levels of pantetheine 4’‐phosphate, isoleucine, and octadec‐5‐enoic acid suggest enhanced mobilization of precursors and cofactors required for membrane remodeling and energy production. Notably, significant accumulation of long‐chain and polyunsaturated acylcarnitines (such as 3,5‐tetradecadiencarnitine), elevated levels of the membrane precursor monoacylglycerol MG(18:2/0:0/0:0), and widespread upregulation of structural and signaling phospholipids—including PS(18:0/18:1), PG(18:1/22:6), and PI(18:0/20:4)—collectively indicate a redirection of lipid metabolism toward the generation of membrane components and lipid‐derived signaling molecules. These metabolic adaptations are consistent with the enhanced membrane dynamics, cytoskeletal reorganization, and vesicular trafficking required for efficient cell migration and invasion. Furthermore, the metabolic profile revealed reduced levels of systemic regulatory factors, including suppressed mineralocorticoid and glucocorticoid signaling, diminished availability of the methyl donor S‐adenosylmethionine, and downregulation of antioxidant metabolites such as hypotaurine (Figure 8B). This metabolic reprogramming suggests PDIA4 promotes invasion by enhancing membrane plasticity, modulating redox balance, and fueling cytoskeletal dynamics. Together, these findings establish that PDIA4 drives a metastasis‐oriented metabolic program underpinning its pro‐invasive functions in TNBC cells. Further pathway enrichment analysis revealed that the three most prominent metabolites—D‐glutamic acid, histidine, and isoleucine—were closely associated with amino acid metabolism, protein digestion and absorption, and ABC transporters. Consequently, these metabolites impacted key biological processes including cellular metabolism, translation, and membrane transport (Figure 8C). In alignment with these findings, Figure 8D shows significant enrichment of three signaling pathways: central carbon metabolism in cancer, biosynthesis of amino acids, and protein digestion and absorption. These results collectively highlight PDIA4 as a central regulator of cancer metabolism and a promising therapeutic target for inhibiting metastasis.

## 3 Discussion

TNBC remains clinically challenging due to its aggressive nature and limited targeted therapeutic options. A key driver of its malignancy is the adaptive reprogramming of proteostasis, which manifests as an acquired dependency on chronic ER stress—a vulnerability termed “ER stress addiction.” Uncontrolled and sustained protein synthesis, a hallmark of rapidly proliferating tumors [19], exposes cancer cells to proteotoxic stress triggered by oncogene activation and heightened secretory activity, leading to the accumulation of unfolded/misfolded proteins in the ER lumen and the induction of chronic ER stress [18, 20]. Within this context, PDIA4, a critical oxidoreductase and molecular chaperone resident in the ER, plays an essential role in maintaining ER proteostasis and enhancing tumor cell adaptability under stress conditions [15]. Emerging evidence positions PDIA4 as an oncogenic driver, with its overexpression documented in malignancies such as multiple myeloma [21] and renal cell carcinoma [22]. Notably, PDIA4 actively promotes tumorigenesis and confers radiotherapy resistance in TNBC [23]. In the present study, we expand the functional landscape of PDIA4 by demonstrating its pleiotropic role as a metabolic orchestrator. Metabolomic profiling revealed that PDIA4 overexpression remodels lipid metabolism toward membrane plasticity—evidenced by accumulation of phospholipids and acylcarnitines—and modulates redox balance via suppression of antioxidants such as hypotaurine. These adaptations are essential for cytoskeletal dynamics and tumor cell survival under migratory stress. Additionally, we identify PDIA4 as a regulator of calcium homeostasis, as its manipulation directly alters total cellular Ca^2+^ levels. Collectively, these findings position PDIA4 as a central hub coordinating precursor supply, lipid metabolic flux, redox balance, and calcium signaling, thereby underscoring its critical importance in tumor metabolic reprogramming and stress adaptation.

Given the central role of PDIA4 in sustaining the malignant phenotype, understanding its upstream regulatory mechanisms is of paramount importance. The Hippo signaling pathway is a well‐established regulator of tumor invasion and metastasis, with its core effectors YAP/TAZ promoting pro‐metastatic gene expression through TEAD4 [23, 24, 25]. Here, we identify a functional dichotomy of TEAD4: under basal conditions, it primarily governs metastatic capacity rather than cell proliferation. Mechanistically, we provide the first direct evidence that TEAD4 binds to the PDIA4 gene promoter and activates its transcription, establishing a direct molecular link between the Hippo pathway and ER proteostasis. This “cross‐pathway regulation” extends the traditional view of the UPR as an autonomous regulator of ER stress and reveals how TNBC cells co‐opt developmental and stress‐adaptation programs to sustain malignancy.

Targeting this axis, we identify the natural polysaccharide THP as a first‐in‐class agent that disrupts the Hippo‐YAP/TEAD4‐PDIA4 axis through a hierarchical mechanism. First, THP activates the MST1‐LATS1 cascade, inducing YAP phosphorylation (Ser127/Ser397), cytoplasmic sequestration, and ubiquitin‐mediated degradation, thereby attenuating YAP/TEAD4 interaction. Second, THP independently downregulates TEAD4 protein levels via an auxiliary, Hippo‐independent pathway, ensuring comprehensive suppression of the axis. This finding aligns with emerging evidence that TEAD4 expression can be modulated independently of YAP/TAZ through mechanisms such as signal pathway crosstalk [26], m6A‐mediated mRNA stabilization [27], or coordinated regulation with YAP [28]. Of note, the observed reduction in TEAD4 chromatin occupancy at the PDIA4 promoter likely reflects both the decrease in total TEAD4 protein abundance and the impaired transcriptional activity of residual TEAD4 due to loss of YAP interaction—collectively contributing to the robust suppression of the axis. The functional consequence is PDIA4 downregulation, which shifts the UPR from an adaptive to a pro‐apoptotic state. While TEAD4 regulates a broad transcriptional network, PDIA4 emerges as a particularly critical effector due to its dual role as both a direct TEAD4 target and a master regulator of ER proteostasis, metabolic reprogramming, and calcium homeostasis. Its downregulation alone is sufficient to convert the tumor's adaptive stress response into a lethal vulnerability, underscoring its functional centrality in the axis. In contrast to canonical ER stress inducers such as tunicamycin, which upregulate protective chaperones, THP suppresses them while activating the PERK‐CHOP axis. This “maladaptive” ER stress response transforms the tumor's survival dependency into a lethal crisis, culminating in apoptosis. Rescue experiments confirmed that re‐expression of PDIA4 reversed the THP‐induced Ca^2+^ decline, establishing causality. PDIA4, also known as calcium‐binding protein 2 (CaBP2), was originally characterized as an ER lumen‐resident calcium‐binding glycoprotein [29, 30, 31]. Given that related family members ERp44 and ERp57 modulate IP3R/SERCA through redox‐dependent mechanisms [32], we speculate that PDIA4 may regulate calcium flux via analogous pathways. Thus, by hierarchically suppressing the YAP/TEAD4‐PDIA4 axis, THP simultaneously cripples the tumor's metastatic machinery, metabolic reprogramming capacity, and stress adaptation mechanisms, converting an adaptive dependency into a lethal vulnerability.

These mechanistic insights translate into potent anti‐metastatic efficacy across multiple preclinical models. THP significantly impaired TNBC cell migration, invasion, and angiogenesis in vitro at concentrations devoid of cytotoxicity (viability > 90% at 24 h). Notably, THP exhibited robust anti‐metastatic effects in patient‐derived organoids, zebrafish xenografts, and immunocompetent mouse models, with efficacy comparable to the chemotherapeutic agent CBP but through a fundamentally distinct mechanism: THP disrupts proteostasis via the YAP/TEAD4‐PDIA4 axis, whereas CBP induces DNA damage. This mechanistic divergence underscores a key principle in cancer therapy: comparable anti‐tumor outcomes can be achieved through engagement of distinct molecular pathways. Notably, and consistent with our previous findings [9], THP exhibited no detectable toxicity in major organs, further supporting its safety for clinical application. These findings highlight the therapeutic advantage of targeting a tumor‐specific stress adaptation pathway rather than core proliferative machinery, which may offer a broader therapeutic window and reduced off‐target effects.

Several questions remain to be addressed. First, while we demonstrate that THP reduces TEAD4 protein levels via a Hippo‐independent pathway, the precise mechanism—whether involving proteasomal degradation, lysosomal turnover, or translational inhibition—requires further investigation. Second, although our data establish PDIA4 as a regulator of calcium homeostasis, the molecular details (e.g., whether PDIA4 modulates IP3R or SERCA activity through redox‐dependent interactions) await biochemical dissection. Third, while our metabolomic analysis revealed a metastasis‐oriented metabolic program driven by PDIA4, the causal contribution of individual metabolite changes to the invasive phenotype warrants validation through targeted interventions. Fourth, although THP can reduce TEAD4 chromatin occupancy at the PDIA4 promoter, the precise mechanism behind this effect—specifically, whether it involves protein degradation or direct transcriptional inhibition—remains to be clarified. Future studies addressing these questions will further elucidate the therapeutic potential and molecular pharmacology of THP.

**FIGURE 9:**
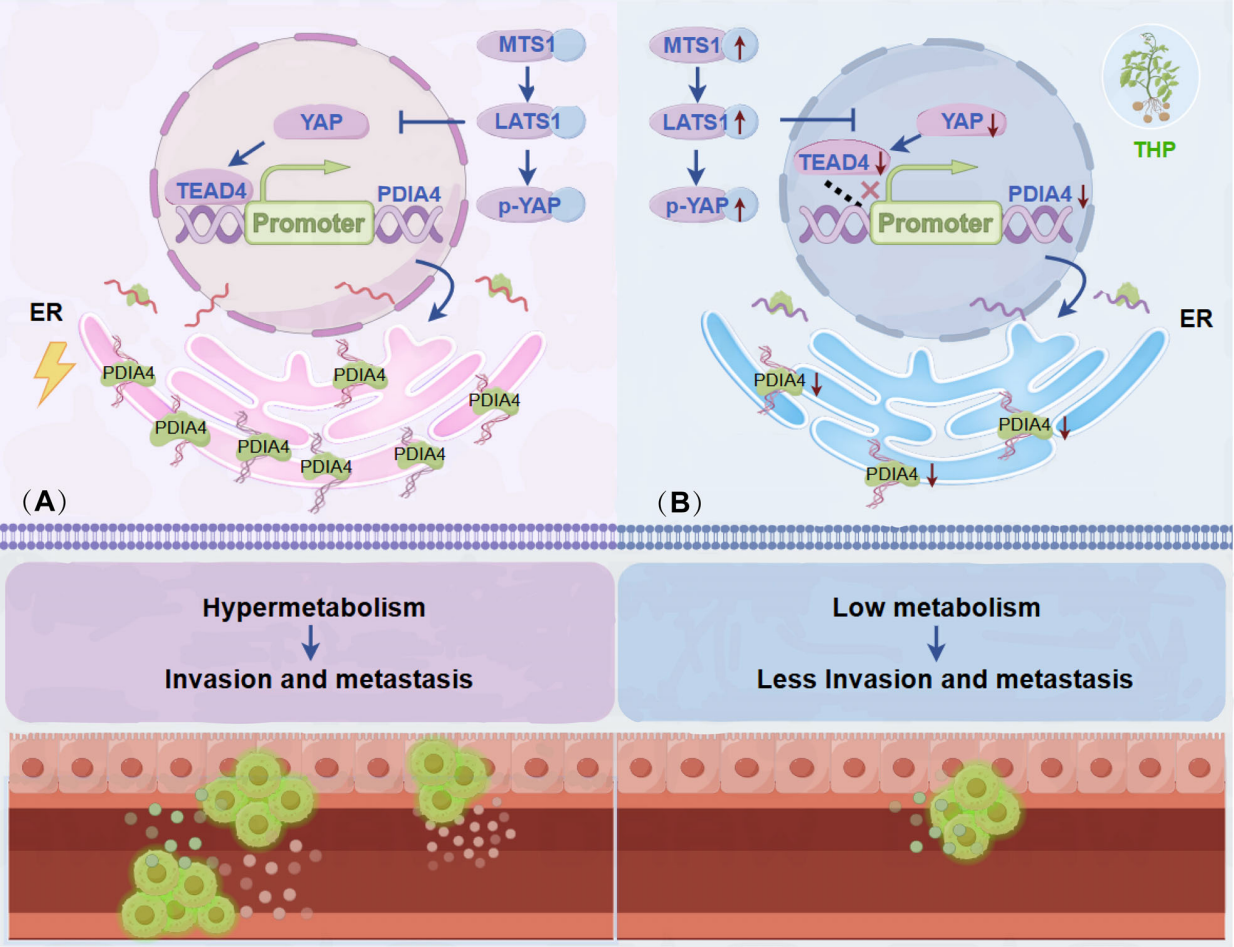
THP rewires the Hippo‐YAP/TEAD4‐PDIA4 axis to shut down cancer cell metabolism and metastasis. (A) Under chronic ER stress, active YAP/TEAD4 complexes bind the PDIA4 promoter, driving PDIA4 transcription and ER accumulation. High PDIA4 levels sustain hyper‐metabolism, protein folding capacity, and invasive behavior. (B) THP activates the MST1–LATS1 cascade, boosting YAP phosphorylation and cytoplasmic retention and degradation. TEAD4‐mediated PDIA4 transcription is silenced, ER proteostasis collapses, metabolism shifts to a low‐activity state, and metastasis is blocked.

In summary, this study reveals the Hippo‐YAP/TEAD4‐PDIA4 axis as a critical and targetable vulnerability in ER‐stress‐addicted TNBC. We establish THP as a first‐in‐class, natural‐product‐based therapeutic agent capable of disrupting this axis through a hierarchical mechanism, thereby converting the adaptive stress response upon which tumors depend into a lethal cellular crisis. These findings not only deepen our understanding of the regulatory mechanisms governing tumor proteostasis but also provide a rationally designed and translationally promising new strategy for treating this most aggressive breast cancer subtype (Figure 9).

## 4 Experimental Section

### 4.1 Preparation and Identification of THP

To ensure consistent chemical composition and biological activity of THP across all experiments, all studies in this research (including cells, zebrafish, and mouse models) were conducted using purified samples from the same batch, designated as THP‐2023‐05‐Batch05. After chemical characterization, aliquots of this batch were stored at –80°C to maintain uniformity and comparability of the material across different experimental setups. The extraction and purification process for this batch of THP was conducted as follows: The leaves and canes of *Tetrastigma hemsleyanum* Diels et Gilg were collected in Shangrao City, Jiangxi Province, China. Monosaccharide standards and lipopolysaccharide (LPS, Sigma, L2880) were used. Plant materials were dried, powdered, and defatted with 95% ethanol (1:15 w/v, 80°C, 2 × 40 min). The residue was extracted with distilled water (1:15 w/v, 95°C, 2 × 60 min), centrifuged (2800 × g, 10 min), and concentrated by rotary evaporation (70°C). Ethanol was added to the concentrate to 70% final concentration, stored at 4°C overnight, and the precipitate was collected by centrifugation, redissolved, de‐ethanized, and freeze‐dried to obtain crude polysaccharides. THP was purified via DEAE‐52 cellulose chromatography (1.6 × 30 cm), eluted with water and NaCl gradients (0.1–0.5 mol/L, 2 mL/min), monitored by phenol‐sulfuric acid assay. For scale‐up, a 2.6 × 50 cm column was used with 200 mg lyophilized powder; the main fraction was desalted by dialysis (3000 Da cutoff), concentrated, and freeze‐dried. Further purification of THP was performed using a Superdex 200 gel filtration column (1.5 × 100 cm, 0.5 mL/min, water elution), yielding the purified polysaccharide THP after lyophilization (Figure S1A–C). To confirm its chemical fingerprint characteristics, a systematic analysis of this batch was conducted. The phenol‐sulfuric acid method, m‐hydroxydiphenyl method, and BCA method were employed to determine its total carbohydrate content as 83.5%, total uronic acid content as 24.73%, and protein content as < 0.01%, with an extraction yield of 67.04% (Table S1 and Figures S1D–F).

### 4.2 Monosaccharide Composition

THP (5 mg) was hydrolyzed with 1 mL of 2 m TFA at 121°C for 2 h. The hydrolysate was dried under nitrogen, washed with methanol, and dried again. This cycle was repeated 2–3 times to ensure complete TFA removal. The residue was dissolved in 1 mL of deionized water, filtered through a 0.22 µm membrane, and analyzed by HPAEC on a Thermo ICS 5000+ system equipped with a Dionex CarboPac PA‐20 column (3 × 150 mm, 10 µm) and PAD detector. A 5 µL aliquot was injected and eluted at 0.5 mL/min With a gradient of mobile phases A (ddH_2_O), B (0.1 m NaOH), and C (0.1 m NaOH + 0.2 m NaAc): 0 min (95:5:0), 26–42 min (85:5:10), 42.1–52 min (60:0:40 → 60:40:0), 52.1–60 min (95:5:0). Data were processed using Chromeleon 7.2 CDS. Monosaccharides were identified by comparing retention times with standards, and molar ratios were calculated via linear regression of standard peak areas vs. concentrations (Figure S2A,B). Following high‐performance anion‐exchange chromatography with pulsed amperometric detection (HPAEC‐PAD) analysis, the molar ratio of monosaccharides was determined as Gal: GlcA: Man: Ara: Rha: Glc = 35.77: 26.95: 20.90: 11.89: 2.95: 1.53 (Table S2).

### 4.3 Homogeneity, Molecular Weight, and Chain Conformation

The analysis was carried out using a U3000 high‐performance liquid chromatography (HPLC) system (Thermo Fisher Scientific, Waltham, MA, USA). The column temperature was precisely regulated at 45°C throughout the experiments. Sample preparation involved dissolving the analyte in a 0.1 m NaNO_3_/0.02% NaN_3_ solution to a concentration of 1 mg/mL, followed by filtration through a 0.45 µm membrane filter. For each chromatographic run, 100 µL of the filtered sample solution was injected into the system. The mobile phase, a 0.1 m NaNO_3_ solution containing 0.02% NaN_3_ (w/w), was pumped through the columns at a constant flow rate of 0.4 mL/min. Detection was performed using an Optilab T‐rEX differential refractive index detector and a DAWN HELEOS II multi‐angle laser light scattering detector, both supplied by Wyatt Technology (Santa Barbara, CA, USA). The analysis revealed a symmetrical single peak in the molecular weight distribution (Figure S3A–D), indicating excellent homogeneity of the sample.

### 4.4 Nuclear Magnetic Resonance (NMR) Spectroscopy Analysis

THP (20 mg) was dissolved in 0.5 mL D_2_O via gentle heating (60°C, 30 min) with intermittent vortexing. After cooling to RT, the solution was transferred to a 5 mm NMR tube. ^1^H, ^1^ ^3^C, COSY, HSQC, HMBC, NOESY, and TOCSY spectra were acquired on Bruker AVANCE NEO 500 M (5 mm PABBO probe, 25°C) and 600 m (PA BBO 600S3 probe, 22°C) spectrometers. Spectra were processed using MestReNova 12.0, with chemical shifts referenced to acetone (δH 2.225, δC 31.07 ppm). The major glycosidic linkage patterns were elucidated (Figure S4A–E).

### 4.5 Cell Culture

Human TNBC MDA‐MB‐231 (Serial: SCSP‐5043) and mouse breast cancer 4T1 cell lines (Serial: SCSP‐5056) were procured from the Cell Bank of the Chinese Academy of Sciences (Shanghai, China). Luciferase‐labeled variants (MDA‐MB‐231‐Luc and 4T1‐Luc) were obtained by transfecting cells with a lentiviral vector containing the Luc gene. MDA‐MB‐231 and MDA‐MB‐231‐Luc cells were maintained in Leibovitz's L‐15 medium (BasalMedia, Shanghai, China) supplemented with 10% fetal bovine serum (FBS) in a CO_2_‐free humidified incubator at 37°C. 4T1 and 4T1‐Luc cells were cultured in RPMI‐1640 medium (BasalMedia, Shanghai, China) supplemented with 10% FBS under standard conditions (37°C, 5% CO_2_, humidified atmosphere). All cell lines were routinely verified to be mycoplasma‐free. Luciferase activity was confirmed prior to experimental use through bioluminescence imaging.

### 4.6 CCK‐8 Assays

Cell viability was evaluated through the CCK‐8 assay. MDA‐MB‐231 and 4T1 cells were plated into 96‐well plates at a density of 2000 cells per well in 200 µL of complete culture medium. After overnight incubation to facilitate cell adhesion, THP was introduced at different concentrations and cultured for 24 and 48 h. Subsequently, 20 µL of CCK‐8 solution was added to each well, and the plates were incubated at 37°C for 3 h. The absorbance at 480 nm was measured by a microplate reader. Cell viability was calculated as a percentage compared to untreated control groups.

### 4.7 Migration and Invasion Assays

MDA‐MB‐231 cells in logarithmic phase were first serum‐starved by culturing in serum‐free medium for 12 h at 37°C with 5% CO_2_. For migration, 200 µL cell suspensions (1 × 10^5^ cells) were added to the upper chamber of Transwell inserts, with 600 µL 10% FBS medium in the lower chamber. For invasion, Transwell inserts were pre‐coated with 1:8 diluted Matrigel, solidified at 37°C for 4 h. After seeding 200 µL cell suspension, the medium in both chambers was replaced with RPMI 1640 containing THP 4 h later. The lower chamber had 20% FBS medium with corresponding THP. Following 24 h incubation at 37°C with 5% CO_2_, chambers were washed with PBS. Upper‐surface cells were removed, and lower‐surface cells were fixed with 4% paraformaldehyde, stained with Crystal Violet (Solarbio, Cat.#G1061, China). Finally, the field of view was randomly selected under the microscope for photography, the number of cells that migrated or invaded the submembrane surface was counted, and the experimental results were analyzed.

### 4.8 Flow Cytometry for Detecting Intracellular Ca^2^^+^ Levels

MDA‐MB‐231 and 4T1 cells were seeded into six‐well plates at 1 × 10^5^ cells/mL. After 24 h, THP was added at a dose of 10ug/mL and administered continuously for 24 h. Cells were loaded with 2–5 µm Fluo‐4 AM (Invitrogen, F14201) in HBSS containing 0.02% Pluronic F‐127 for 30–60 min at 37°C in the dark. Excess probe was removed by three washes with HBSS. Cells were then resuspended in calcium‐free HBSS. Samples were pre‐incubated at 37°C for 5–15 min before the addition of 1 mm CaCl_2_. Fluorescence intensity was immediately measured using a flow cytometer (BD FACSCalibur) with excitation at 488 nm and emission detection at 530/30 nm. Baseline fluorescence was recorded for 15–30 s, followed by the addition of stimuli through the injection port and continuous data acquisition for 3–5 min. Data were analyzed using FlowJo software. Peak MFI was quantified for each sample and used for statistical analysis.

### 4.9 Ca^2^^+^ Assay

MDA‐MB‐231 and 4T1 cells were seeded into 96‐well plates at a density of 1 × 10^5^ cells/mL. After 12 h of incubation, cells were treated with THP for 24 h. Subsequently, cells were harvested, transferred to centrifuge tubes, and centrifuged at 1000 rpm for 5 min. The cell pellet was resuspended in 0.3–0.5 mL of deionized water, and cells were disrupted by sonication or manual grinding. After disruption, the samples were centrifuged at 2000 rpm for 10 min, and the supernatants were collected for Ca^2^^+^ concentration measurement using a 96T Calcium Assay Kit (Nanjing Jiancheng Bioengineering Institute, C004‐2‐1, Nanjing, China) according to the manufacturer's protocol. Absorbance was measured at 610 nm using a microplate reader.

### 4.10 RNA Sequencing

MDA‐MB‐231 cells (3 × 10^4^ cells/mL) were seeded, treated with THP for 24 h after 12 h incubation. Total RNA was extracted, with purity (OD_260_/OD_280_: 1.8‐2.2, OD_260_/OD_230_≥2.0) and integrity (RIN≥7) verified by spectrophotometer and Bioanalyzer. Poly (A)+ mRNA was enriched, fragmented (150–200 bp), reverse‐transcribed, and libraries were prepared via end repair, adapter ligation, PCR, and bead purification. Sequencing was performed on Illumina platforms (paired‐end, Q30 ≥ 85%). Reads were quality‐controlled by FastQC, aligned with STAR, and quantified by Kallisto. DESeq2 identified differentially expressed genes (|log_2_FC|≥ 1, FDR< 0.05) for GO/KEGG analysis, with optional qRT‐PCR validation.

### 4.11 Transmission Electron Microscopy

Following 24 h of treatment with THP, cell pellets were collected and fixed overnight at 4°C in 2.5% glutaraldehyde. Samples were then rinsed sequentially with phosphate buffer and 1% osmium tetroxide, dehydrated through a graded acetone series, embedded in epoxy resin, and sectioned into ultrathin slices (70–90 nm). Sections were mounted on copper grids and imaged using an H7500 transmission electron microscope (Hitachi, Japan) at an accelerating voltage of 80 kV.

### 4.12 Western Blot Assays

Equal amounts of protein were separated by 10% sodium dodecyl sulfate‐polyacrylamide gel electrophoresis (SDS‐PAGE, Beyotime, China) and transferred to polyvinylidene fluoride (PVDF) membranes (Millipore, USA). At room‐temperature, block in 5% skimmed milk diluted with TBST for 1 h, then wash with TBST three times, and then incubate overnight with the primary antibody diluted with the primary antibody dilution solution at 4°C: GAPDH (1:5,000, Proteintech, Cat.#60004‐1‐Ig, USA), LATS1 (1:1000, Cell Signaling Technology, Cat.#3682, USA), MST1 (1:1,000, Cell Signaling Technology, Cat.#3952, USA), YAP (1:5000, Proteintech, Cat.#13584‐1‐AP, USA), p‐YAP1 (Ser397) (1:2,000, Proteintech, Cat.#29018‐1‐AP, USA), p‐YAP1 (Ser127) (1:10 000, abcam, Cat.#ab76252, USA), Vimentin (1:2000, Proteintech, Cat.#10366‐1‐AP, USA), TEAD4 (1:2000, Proteintech, Cat.# 12418‐1‐AP, USA), PDIA4 (1:2000, Proteintech, Cat.#14712‐1‐AP, USA), Bip (1:2000, Proteintech, Cat.#11587‐1‐AP, USA), p‐PERK (1:5000, Proteintech, Cat.#82534‐1‐RR, USA), p‐IRE1 (1:5000, abcam, Cat.#ab124945, USA), cleaved ATF6 (1:5000, Proteintech, Cat.#24169‐1‐AP, USA), and CHOP (1:2000, Proteintech, Cat.#15204‐1‐AP, USA), β‐actin (1:1000, Cell Signaling Technology, Cat.#4970, USA). After three 10 min TBST washes, membranes were incubated with HRP‐conjugated secondary antibodies (1:10 000, Biosharp, China) in 5% skimmed milk for 1 h at room‐temperature, followed by three additional TBST washes. Protein bands were visualized using an enhanced chemiluminescence (ECL) substrate and imaged using ImageJ software (NIH, USA).

### 4.13 Pan‐Cancer Analysis of TEAD4 and PDIA4 Expression

We downloaded the uniformly normalized pan‐cancer dataset TCGA TARGET GTEx (PANCAN, N = 19131, G = 60499) from the UCSC database (https://xenabrowser.net/). Expression data for ENSG00000197905 (TEAD4) and ENSG00000155660 (PDIA4) across samples were extracted, with samples filtered to include only those from solid tissue normal, primary solid tumor, primary tumor, normal tissue, primary blood‐derived cancer‐bone marrow, and primary blood‐derived cancer‐peripheral blood. Each expression value was subjected to log2 (x+0.001) transformation, and cancer types with fewer than three samples were excluded, resulting in expression data for 34 cancer types. Using R software (version 3.6.4), we calculated expression differences between normal and tumor samples in each cancer type, with statistical significance assessed by unpaired Wilcoxon Rank Sum and Signed Rank Tests. TEAD4 was significantly upregulated in 26 cancers, such as BRCA (Tumor: 3.63 ± 0.87 vs. Normal: 3.09 ± 0.74, p = 4.6 × 10^−^^2^ ^7^). PDIA4 was significantly upregulated in 31 cancers, including GBM (Tumor: 6.69 ± 0.77 vs. Normal: 3.26 ± 1.32, p = 1.1 × 10^−^^8^ ^7^), GBMLGG (Tumor: 5.71 ± 0.94 vs. Normal: 3.26 ± 1.32, p = 1.5 × 10^−^^2^ ^4^ ^1^), and BRCA (Tumor: 6.75 ± 0.66 vs. Normal: 5.34 ± 0.69, p = 4.4 × 10^−^^1^ ^1^ ^7^).

### 4.14 Prognostic Analysis of TEAD4 and PDIA4 Expression in Pan‐Cancer

Using the maxstat package (version 0.7‐25) in R, an optimal cutoff value of 1.3846 was determined for both ENSG00000197905 (TEAD4) and ENSG00000155660 (PDIA4), with group size constraints set to include ≥ 25% and ≤ 75% of the total cohort. Patients were stratified into high‐ and low‐expression groups based on this cutoff. Survival curves were generated using the survfit function from the survival package, and log‐rank test analysis revealed significant prognostic differences for both genes: high TEAD4 expression predicted worse overall survival (p = 4.2 × 10^−^^2^ ^1^), while high PDIA4 expression was associated with even more pronounced survival disparity (p = 1.7 × 10^−^^5^ ^9^).

### 4.15 PDIA4 Genomic Heterogeneity and Correlation with Tumor Mutation Burden

The uniformly normalized TCGA Pan‐Cancer (PANCAN) dataset (N = 10535 samples, G = 60 499 genes) was downloaded from the UCSC Xena Browser (https://xenabrowser.net/). Expression data for ENSG00000155660 (PDIA4) were extracted, and samples were filtered to include only those from Primary Blood Derived Cancer‐Peripheral Blood and Primary Tumor. Additionally, Level‐4 Simple Nucleotide Variation (SNV) datasets for all TCGA samples, processed using MuTect2 (https://doi.org/10.1038/nature08822), were downloaded from the GDC Data Portal (https://portal.gdc.cancer.gov/). Tumor mutation burden (TMB) was calculated for each sample using the tmb function in the R package maftools (version 2.8.05). PDIA4 expression values were log2‐transformed (log2[x+0.001]), and cancer types with fewer than three samples were excluded, resulting in 37 cancer types for analysis. Pearson correlation coefficients were computed between PDIA4 expression and TMB in each cancer type. Significant positive correlations were observed in 11 cancer types, exemplified by BRCA (N = 981, R = 0.066, p = 0.037).

### 4.16 Plasmid Design and Lentiviral Infection

The GV367 vector (Ubi‐MCS‐SV40‐EGFP‐IRES‐puromycin) was used to construct PDIA4 overexpression and control plasmids, while the GV248 construct (hU6‐MCS‐Ubiquitin‐EGFP‐IRES‐puromycin) was employed for shRNA generation. Human PDIA4 overexpression plasmids and shRNA targets (shPDIA4‐1: 5'‐CCAAGAAGTACAAGGGCCAAA‐3'; shPDIA4‐2: 5'‐GCAAGGTGTCAAACG ATGCTA‐3'; shPDIA4‐3: 5'‐CCTGAGAGAAGATTACAAATT‐3') were designed and constructed by Gemma Gene Co., Ltd. (China).

### 4.17 siRNA Transfection

TEAD4‐targeting siRNAs were synthesized by Ribobio Co., Ltd. (Guangzhou, China). The sequences of the four siRNAs were as follows: TEAD4 siRNA#1 Sense: 5'‐CAGACCUCAACACCAACAUTT‐3', TEAD4 siRNA#1 Antisense: 5'‐AUGUUG GUGUUGAGGUCUGTT‐3'; TEAD4 siRNA#2 Sense: 5'‐GACAGAGUAUGCUC GCUAUTT‐3', TEAD4 siRNA#2 Antisense: 5'‐AUAGCGAGCAUACUCUGUCTT ‐3'; TEAD4 siRNA#3 Sense: 5'‐CUCCCUGAGAAGUACAUGATT‐3', TEAD4 siRNA#3 Antisense: 5'‐UCAUGUACUUCUCAGGGAGTT‐3'; TEAD4 siRNA#4 Sense: 5'‐GCAUUGCCUAUGUCUUUGATT‐3', TEAD4 siRNA#4 Antisense: 5'‐UC AAAGACAUAGGCAAUGCTT‐3'. Transfected cells were harvested 48–72 h post‐transfection, and the knockdown efficiency of TEAD4 was validated by RT‐qPCR and Western blot.

### 4.18 RNA Extraction and RT‐qPCR

Total RNA was extracted using the RNA Extraction Kit (Vazyme, CL111‐01, China). Complementary DNA (cDNA) was synthesized from 1 µg of total RNA per sample using the Reverse Transcription Kit (Vazyme, R123‐01, China). Quantitative real‐time PCR (RT‐qPCR) was performed on a qTower3 G PCR System (Analytik Jena, Germany) to quantify mRNA expression levels. The primer sequences used in this study were as follows: PDIA4: Forward: 5'‐TTGTTGGCGTAGATTTGGCT‐3' Reverse: 5'‐TGCTCAGTGGCAGCTCTCAC‐3'; TEAD4: Forward: 5'‐GGCAGACC TCAACACCAACATC‐3', Reverse: 5'‐AGCAGACCTTCGTGGAGCAG‐3'; HSPA5: Forward: 5'‐CTGTCCAGGCTGGTGTGCTCT‐3', Reverse: 5'‐CTTGGTAGGCACCACTGTGTTC‐3'; GAPDH (internal control): Forward: 5'‐GGTGAAGGTCGGAGTCAACG‐3'; Reverse: 5'‐TGGGTGGAATCATATTGGAACA‐3'.

### 4.19 ChIP Assay

To investigate whether TEAD4 binds to the promoters of PDIA4 and HSPA5, we performed two independent chromatin immunoprecipitation (ChIP) assays. For each gene, potential TEAD4 binding motifs were predicted using the JASPAR Core database (https://jaspar.genereg.net/) in combination with publicly available ChIP‐seq data from the Cistrome Data Browser (http://cistrome.org/db/#/). Three high‐confidence regions suitable for primer design were selected in each promoter for experimental validation. For the PDIA4 promoter, the following primer pairs were used to amplify immunoprecipitated DNA fragments: Primer 1 (forward: 5ʹ‐CGGGAGGCTGAGACAGTAGA‐3ʹ, reverse: 5ʹ‐CTATGAATGTGGGCCACCAG‐3ʹ); Primer 2 (forward: 5ʹ‐TCTTTTCCCTATTTGCCTTCT C‐3ʹ, reverse: 5ʹ‐AGCTTGAGGTTTTCCCTCTTG‐3ʹ); Primer 3 (forward: 5ʹ‐GCAC TTCAAGAGCCTTCTTCC‐3ʹ, reverse: 5ʹ‐CCTGTTCTTCAAAAAGACAAGTCA ‐3ʹ). For the HSPA5 promoter, the corresponding high‐confidence regions were examined with the following primers: Primer 1 (forward: 5ʹ‐GCTTTCTCTTCCCAGCTTCC‐3ʹ, reverse: 5ʹ‐CGACCTGTGAGCAACGAAC‐3ʹ); Primer 2 (forward: 5ʹ‐CTGCCCATCCTTTCCCTCT‐3ʹ, reverse: 5ʹ‐GGCATTATCAAGACGATTTTCG‐3ʹ); Primer 3 (forward: 5ʹ ‐TCATATCAGAGCTTTGCCCTTT‐3ʹ, reverse: 5ʹ‐GAGGTGGAGGTTGCAGTGAG‐3ʹ). ChIP assays were performed using a Chromatin Immunoprecipitation Kit (Vazyme, HD101‐01, China). Briefly, MDA‐MB‐231 cells were seeded at a density of 3 × 10^4^ cells per well and cultured for 12 h. Cells were then divided into three groups: IgG (negative control), TEAD4 (positive control), and THP‐treated (experimental). The THP‐treated group was incubated with THP for 24 h prior to cell harvesting. Subsequent steps were strictly performed according to the manufacturer's instructions.

### 4.20 Dual‐Luciferase Reporter Assay

The promoter sequences of PDIA4—Including the control plasmid, wild‐type full‐length, site 1 mutant, and site 2 mutant—were individually cloned into a firefly luciferase reporter plasmid. Each construct was co‐transfected into MDA‐MB‐231 cells along with a Renilla luciferase vector. After 24 h, luciferase activity was measured using the Dual‐Luciferase Reporter Assay System (Beyotime, Cat. #RG029M, Shanghai, China) following the manufacturer's protocol.

### 4.21 Generation of TNBC Patient‐Derived Organoids (PDOs)

Surgical TNBC specimens were placed in sterile 10 cm dishes, rinsed with tissue dissociation medium (Hangzhou AimingMed Technologies Co., China), and finely minced into small fragments of 1–2 mm^3^. Digestion was terminated when cell clusters were <150 µm. Cells were filtered (100 µm), centrifuged (300 g, 5 min), and resuspended in Matrigel. 30 µL droplets were plated in 24‐well plates and solidified (15 min, 37°C). Complete medium (DMEM/F12 with B‐27, EGF, FGF, HGF, and insulin‐transferrin‐selenium) was added (600 µL/well). Cultures were maintained at 37°C/5% CO_2_ with 50% medium changes every 48 h. Organoids were passaged at 1:3 using dissociation reagent upon cystic structure formation (7–14 days). All procedures followed biosafety standards with regular mycoplasma/STR checks. The study was approved by the Ethics Committee of Fujian Medical University Affiliated First Hospital (Approval No. [2024]710).

### 4.22 Zebrafish Culture and Experiments

AB strain zebrafish (Guangdong Longseek Testing Co., Ltd.) were used in this study. Healthy 48 h post‐fertilization (hpf) embryos were selected and randomly placed onto agar plates. Five experimental groups were established: MOD, PTX (1 µg/mL), and THP at concentrations of 10, 20, and 40 µg/mL. mCherry‐labeled MDA‐MB‐231 cells were microinjected into the yolk sac of each embryo using a micromanipulator‐based injection system. After injection, embryos were incubated at 28.5 °C for 1 h. Embryos with successful tumor cell implantation were then selected under a fluorescence microscope and treated with the corresponding drugs. All groups were maintained at 28.5°C. Tumor cell metastasis was evaluated at 48 h post‐treatment by fluorescence imaging. The migration distance of metastatic tumor cells from the primary injection site was measured using ImageJ software. For each zebrafish, the migration distance was quantified and compared across treatment groups. The zebrafish experiment protocol was reviewed and approved by the Animal Welfare and Ethics Committee of Guangdong LongSeek Testing Co., Ltd., and complies with the principles of animal protection, animal welfare, and ethics, as well as relevant national regulations.

### 4.23 Mouse Experiments

Healthy female BALB/c mice and nude mice were obtained from the Experimental Animal Center of Fujian Medical University (the license number: SYXK (MIN) 2020‐0005, and the ethical approval number of the animal model study is IACUC FJMU 2024‐Y‐2415). All animals were housed in standard conditions (25 ± 3°C, 60 ± 5% humidity, and a 12 h light/dark cycle) with free access to complete pellet feed and water. All methods were carried out in accordance with relevant guidelines and regulations. All animals were acclimated for 7 days before the experiment. We recorded body weight every other day. All mice were modeled by tail vein inoculation and randomly grouped (the inoculation amount of 4T1 cells was 1 × 10^6^ cells per BALB/c mouse, and the inoculation amount of MDA‐MB‐231 cells was 3 × 10^6^ cells per nude mouse). THP was administered by gavage at a dose of 20 mg/kg daily, and carboplatin (CBP) was treated once a week at a dose of 100 mg/kg.

### 4.24 Hematoxylin‐eosin (HE) Staining

Major organ tissues (heart, liver, spleen, and kidney) isolated from mice were fixed in 4% paraformaldehyde, embedded in paraffin, and sectioned at a thickness of 4 µm. The sections were deparaffinized, rehydrated, and stained with HE. After staining, the sections were dehydrated, cleared, and mounted with neutral balsam. Pathological changes were observed and photographed under an optical microscope.

### 4.25 Immunohistochemical (IHC) staining

For IHC analysis, paraffin‐embedded tissue sections (4 µm) were subjected to antigen retrieval by heating in EDTA buffer (pH 8.0) under high pressure for 15 min. After cooling to room‐temperature, the sections were incubated overnight at 4°C with primary antibodies against TEAD4 (1:250) and PDIA4 (1:500) in a humidified chamber. The sections were then washed three times with PBS (5 min each) and incubated with a biotinylated secondary antibody (1:200) for 1 h at room‐temperature. Immunoreactivity was visualized using a DAB chromogenic kit in the dark; development was monitored under a microscope and terminated by rinsing with distilled water. Finally, the sections were counterstained (if applicable), dehydrated through a graded ethanol series, cleared, and mounted with neutral balsam. Images were captured using a digital scanner.

### 4.26 Metabolomics Detection of Tumor Tissues

Metabolomic analysis was performed by Shanghai Bioprofile Technology Co., Ltd. (Shanghai, China). For xenograft mouse models, lung metastatic tumor tissues from each group were collected, lyophilized, and ground in precooled methanol:acetonitrile: water (2:2:1, v/v/v). The mixture was sonicated in an ice bath, incubated at ‐20°C, and centrifuged to collect supernatants, which were then vacuum‐dried. UHPLC‐MS/MS analysis was performed on an HSS T3 column using a gradient of 0.1% formic acid in water/acetonitrile. Mass spectra were acquired in positive and negative modes on a Q‐Exactive Plus, with full MS at 70 000 resolution and dd‐MS^2^ at 17 500 resolution. The raw data were processed using MS‐DIAL software, and metabolite structural identification was performed by matching precise mass numbers (mass tolerance < 10 ppm) and secondary mass spectra (mass tolerance < 0.01 Da), with searches conducted against public databases such as HMDB, MassBank, and GNPS, as well as the in‐house Bioprofile metabolite database (BP‐DB). Metabolite features were filtered, and only those with a non‐zero value proportion exceeding 50% in at least one group were retained for subsequent analysis. Multivariate analyses (PCA, PLS‐DA, OPLS‐DA) with Pareto scaling were conducted in R, and significant metabolites were defined by VIP > 1.0 and *p* < 0.05. KEGG pathway enrichment was performed using Fisher's exact test with FDR correction (*p* < 0.05) to identify perturbed metabolic pathways.

### 4.27 Statistical Analysis

Statistical analyses were performed using GraphPad Prism 8. For comparisons between two groups, an unpaired two‐tailed Student's *t*‐test was applied. Differences across multiple groups were evaluated by one‐way ANOVA followed by Dunnet's or Tukey's post hoc tests, as appropriate. Data are presented as mean ± standard deviation (SD). Mouse survival rates were analyzed via Kaplan–Meier survival curves with log‐rank tests. Statistical significance was defined as ^*^ *p* < 0.05, ^**^ *p* < 0.01, and ^***^ *p* < 0.001.

## Author Contributions

Y.Sh. conceptualized, optimized, and performed the experiments; analyzed and interpreted the data; and wrote the manuscript. W.S. performed and analyzed the experiments. Y.Zh., Y.L., and H.S. analyzed data and contributed to data interpretation. J.L. and H.Zh. contributed to patient‐derived breast cancer organoid experiments, as well as data analysis and interpretation. Zh.D. contributed to experimental design and data interpretation. W.J. acquired funding and contributed to data analysis. L.W. conceptualized the study, acquired funding, contributed to data interpretation, and wrote the manuscript. L.W. supervised Y.Sh. and W.S. All authors approved the final version of the manuscript.

## Conflicts of Interest

The authors declare no conflicts of interest.

## Supporting information




**Supporting File**: advs75063‐sup‐0001‐SuppMat.docx.

## Data Availability

The data that support the findings of this study are available on request from the corresponding author.

## References

[1] Y. Li , Q. Zhang , J. Yang , et al., “Metformin Combined with Glucose Starvation Synergistically Suppress Triple‐Negative Breast Cancer by Enhanced Unfolded Protein Response,” Biochemical and Biophysical Research Communications 675 (2023): 146–154, 10.1016/j.bbrc.2023.07.029. 37473529

[2] J. Calahorra , J. L. Blaya‐Canovas , O. Castellini‐Perez , et al., “Unlocking the Effective Alliance of Beta‐Lapachone and Hydroxytyrosol against Triple‐Negative Breast Cancer Cells,” Biomedicine & Pharmacotherapy 174 (2024): 116439. 38518601 10.1016/j.biopha.2024.116439

[3] S. U. Khan , K. Fatima , U. Singh , P. P. Singh , and F. Malik , “Small Molecule ‘4ab’–Induced Autophagy and Endoplasmic Reticulum Stress–Mediated Death of Aggressive Cancer Cells Grown Under Adherent and Floating Conditions,” Medical Oncology 40, no. 4 (2023): 121, 10.1007/s12032-023-01963-5. 36939976

[4] C. Y. Wang , H. J. Jang , Y. K. Han , et al., “Alkaloids from Tetrastigma Hemsleyanum and Their Anti‐Inflammatory Effects on LPS‐Induced RAW264.7 Cells,” Molecules (Basel, Switzerland) 23, no. 6 (2018): 1445. 29899226 10.3390/molecules23061445 PMC6099609

[5] X. Chen , M. Weng , M. Lan , et al., “Superior Antibacterial Activity of Sulfur‐Doped G‐C_3_N_4_ Nanosheets Dispersed by Tetrastigma Hemsleyanum Diels & Gilg's Polysaccharides‐3 Solution,” International Journal of Biological Macromolecules 168 (2021): 453–463, 10.1016/j.ijbiomac.2020.11.155. 33275975

[6] B. Zhu , C. Qian , F. Zhou , et al., “Antipyretic and Antitumor Effects of a Purified Polysaccharide from Aerial Parts of Tetrastigma Hemsleyanum,” Journal of Ethnopharmacology 253 (2020): 112663, 10.1016/j.jep.2020.112663. 32045682

[7] T. Ji , W. W. Ji , J. Wang , et al., “A Comprehensive Review on Traditional Uses, Chemical Compositions, Pharmacology Properties and Toxicology of Tetrastigma Hemsleyanum,” Journal of Ethnopharmacology 264 (2021): 113247, 10.1016/j.jep.2020.113247. 32800929 PMC7422820

[8] T. Wu , X. Wang , H. Xiong , et al., “Bioactives and Their Metabolites from Tetrastigma Hemsleyanum Leaves Ameliorate DSS‐Induced Colitis via Protecting the Intestinal Barrier, Mitigating Oxidative Stress, and Regulating the Gut Microbiota,” Food & Function 12, no. 23 (2021): 11760–11776, 10.1039/D1FO02588K. 34747421

[9] Y. Shang , M. Zhao , S. Chen , et al., “Tetrastigma Hemsleyanum Polysaccharide Combined with Doxorubicin Promote Ferroptosis and Immune Function in Triple‐Negative Breast Cancer,” International Journal of Biological Macromolecules 275, no. Pt 1 (2024): 133424, 10.1016/j.ijbiomac.2024.133424. 38945330

[10] X. Li , P. Yang , T. Wang , et al., “Positive Feedback Regulation between USP8 and Hippo/YAP Axis Drives Triple‐Negative Breast Cancer Progression,” Cell Death & Disease 17, no. 1 (2026): 98, 10.1038/s41419-025-08356-8. 41565619 PMC12830590

[11] Z. Zhu , R. Ding , W. Yu , Y. Liu , Z. Zhou , and C.‐Y. Liu , “YAP/TEAD4/SP1‐Induced VISTA Expression as a Tumor Cell–Intrinsic Mechanism of Immunosuppression in Colorectal Cancer,” Cell Death & Differentiation 32, no. 5 (2025): 911–925, 10.1038/s41418-025-01446-2. 39875519 PMC12089306

[12] M. Kaur , R. F. Mungurere , N. Mitinje , G. K. Sethi , A. S. Kaur , and A. Mishra , “Hippo‐YAP/TAZ Signaling in Gastric Cancer: Molecular Pathogenesis and Emerging Therapeutic Horizons,” Medical Oncology 43, no. 3 (2026): 147, 10.1007/s12032-026-03246-1. 41656414

[13] Y. Guo , G. Song , H. Zou , et al., “PARP1‐Mediated PARylation of TEAD4 Stabilizes the YAP1–TEAD4 Complex and Promotes Growth and Immune Evasion in Breast Cancer Cells,” Science Signaling 18, no. 909 (2025): adx2532, 10.1126/scisignal.adx2532. 41118450

[14] C. Hetz , K. Zhang , and R. J. Kaufman , “Mechanisms, Regulation and Functions of the Unfolded Protein Response,” Nature Reviews Molecular Cell Biology 21, no. 8 (2020): 421–438, 10.1038/s41580-020-0250-z. 32457508 PMC8867924

[15] G. Twito , F. A. Abu Abayed , A. Gilad , et al., “Non‐Genetic Inactivation of Caspase‐3 and p53 Increases Cancer Cell Fitness by PDIA4 Redistribution,” Oncogene 44, no. 47 (2025): 4565–4575, 10.1038/s41388-025-03606-7. 41120732 PMC12623246

[16] Z. Tu , C. Wang , Q. Hu , et al., “Protein Disulfide‐Isomerase A4 Confers Glioblastoma Angiogenesis Promotion Capacity and Resistance to Anti‐Angiogenic Therapy,” Journal of Experimental & Clinical Cancer Research 42, no. 1 (2023): 77, 10.1186/s13046-023-02640-1. 36997943 PMC10061982

[17] Z. W. Ye , J. Zhang , M. Aslam , A. Blumental‐Perry , K. D. Tew , and D. M. Townsend , “Protein Disulfide Isomerase Family Mediated Redox Regulation in Cancer,” Advances in Cancer Research 160 (2023): 83–106. 37704292 10.1016/bs.acr.2023.06.001 PMC10586477

[18] X. Li , C. Lebeaupin , A. Kadianaki , et al., “Activated ATF6α Is a Hepatic Tumour Driver Restricting Immunosurveillance,” Nature 651 (2026): 796–807. 41639449 10.1038/s41586-025-10036-8 PMC12999494

[19] D. Hanahan and R. A. Weinberg , “Hallmarks of Cancer: the Next Generation,” Cell 144, no. 5 (2011): 646–674, 10.1016/j.cell.2011.02.013. 21376230

[20] G. M. Renna , A. Cherubini , E. Varone , S. Germani , A. Marrazza , and E. Zito , “ER Proteostasis Meets Mitochondrial Function: Contact Sites as Hubs of Communication and Therapeutic Targets,” The FEBS Journal (2026), 10.1111/febs.70431. PMC13580312 41609403

[21] W. Yu , J. Zhan , Y. Wang , et al., “Single‐Cell Transcriptomics Identifies PDIA4 as a Marker of Progression and Therapeutic Vulnerability in Multiple Myeloma,” Journal of Translational Medicine 23, no. 1 (2025): 1136, 10.1186/s12967-025-07098-7. 41121130 PMC12538736

[22] L. Kang , D. Wang , T. Shen , et al., “PDIA4 Confers Resistance to Ferroptosis via Induction of ATF4/SLC7A11 in Renal Cell Carcinoma,” Cell Death & Disease 14, no. 3 (2023): 193, 10.1038/s41419-023-05719-x. 36906674 PMC10008556

[23] J. Tao , C. Xue , M. Cao , et al., “Protein Disulfide Isomerase Family Member 4 Promotes Triple‐Negative Breast Cancer Tumorigenesis and Radiotherapy Resistance Through the JNK Pathway,” Breast Cancer Research 26, no. 1 (2024): 1, 10.1186/s13058-023-01758-6. 38167446 PMC10759449

[24] D. Li , A. Wang , X. Wang , et al., “The TEAD4–DYNLL1 Axis Accelerates Cell Cycle Progression and Augments Malignant Properties of Lung Adenocarcinoma Cells,” European Journal of Medical Research 30, no. 1 (2025): 221, 10.1186/s40001-025-02500-y. 40170083 PMC11959721

[25] Z. Song , S. Gui , X. Rao , G. Zhang , Y. Cheng , and T. Zeng , “TAZ/NRF2 Positive Feedback Loop Contributes to Proliferation in Bladder Cancer through Antagonistic Ferroptosis,” Cell Death Discovery 11, no. 1 (2025): 208, 10.1038/s41420-025-02506-9. 40301305 PMC12041353

[26] H. Huh , D. Kim , H.‐S. Jeong , and H. Park , “Regulation of TEAD Transcription Factors in Cancer Biology,” Cells 8, no. 6 (2019): 600, 10.3390/cells8060600. 31212916 PMC6628201

[27] Y.‐Q. Wang , D.‐H. Wu , D. Wei , et al., “TEAD4 is a Master Regulator of High‐Risk Nasopharyngeal Carcinoma,” Science Advances 9, no. 1 (2023): add0960, 10.1126/sciadv.add0960. PMC9821866 36608137

[28] T. Dong , L. Liu , Y. You , et al., “WISP1 Inhibition of YAP Phosphorylation Drives Breast Cancer Growth and Chemoresistance via TEAD4 Activation,” Anti‐Cancer Drugs 36, no. 3 (2025): 157–176, 10.1097/CAD.0000000000001687. 39774151 PMC11781553

[29] M. Vedi , J. R. Smith , G. Thomas , et al., “2022 updates to the Rat Genome Database: a Findable, Accessible, Interoperable, and Reusable (FAIR) Resource,” Genetics 224, no. 1 (2023): iyad042. 36930729 10.1093/genetics/iyad042 PMC10474928

[30] C. UniProt , “UniProt: a Worldwide Hub of Protein Knowledge,” Nucleic Acids Research 47, no. D1 (2019): D506–D515. 30395287 10.1093/nar/gky1049 PMC6323992

[31] P. V. S. Oliveira , T. C. De Bessa , and F. R. M. Laurindo , “Endoplasmic Reticulum Redoxome: Protein Folding and beyond,” Biochemistry 65 (2026): 1–30, 10.1021/acs.biochem.5c00527. 41384921 PMC12781119

[32] C. Appenzeller‐Herzog and T. Simmen , “ER‐Luminal Thiol/Selenol‐Mediated Regulation of Ca^2^ ^+^ Signalling,” Biochemical Society Transactions 44, no. 2 (2016): 452–459, 10.1042/BST20150233. 27068954

