# Supplementary material for: Tetrastigma Hemsleyanum Polysaccharide Suppresses Triple‐Negative Breast Cancer by Disrupting the Hippo‐YAP/TEAD4‐PDIA4 Axis and Endoplasmic Reticulum Stress Adaptation
Source: Adv Sci (Weinh). 2026 Apr 2;13(34):e19006. doi: 10.1002/advs.202519006 (PMC13285156; doi:10.1002/advs.202519006)
Supplement: Supplementary file 1 — Supporting File: advs75063‐sup‐0001‐SuppMat.docx. [file ADVS-13-e19006-s001.docx]

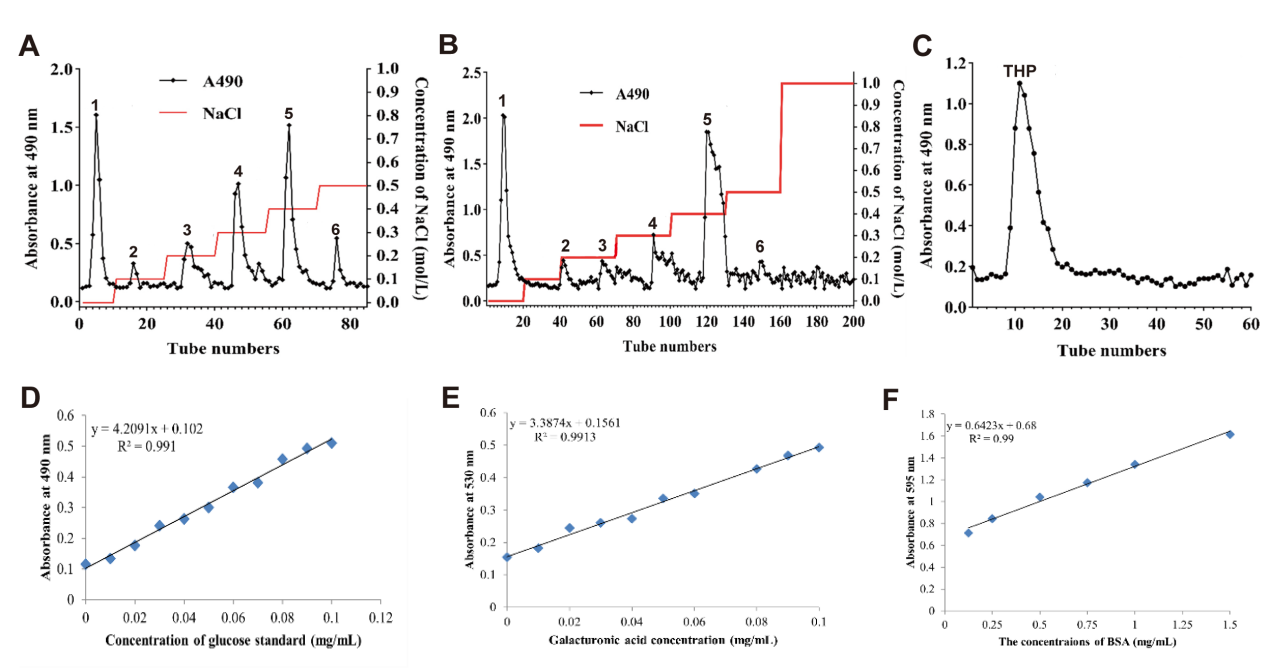


**Figure S1.** Purification and content analysis of THP. A) Elution curve of THP on DEAE-52 cellulose column (1.6 × 30 cm). B) Elution curve of THP on DEAE-52 cellulose column (2.6 × 50 cm). C) Elution curve of THP-5 on Superdex 200 gel filtration column (1.5 × 100 cm). D) Standard curve for total carbohydrate content determination in THP (using glucose as standard). E) Standard curve for total uronic acid content determination in THP (using galacturonic acid as standard). F) Standard curve for THP protein content determination (using BSA as standard).


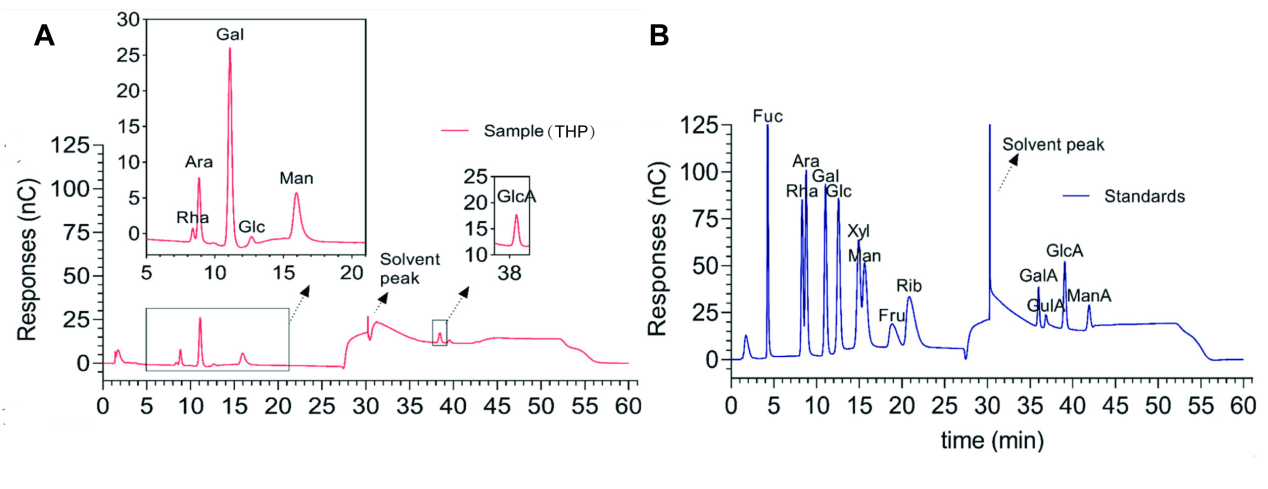


**Figure S2.** Monosaccharide composition analysis. A) THP. B) Monosaccharide standards.


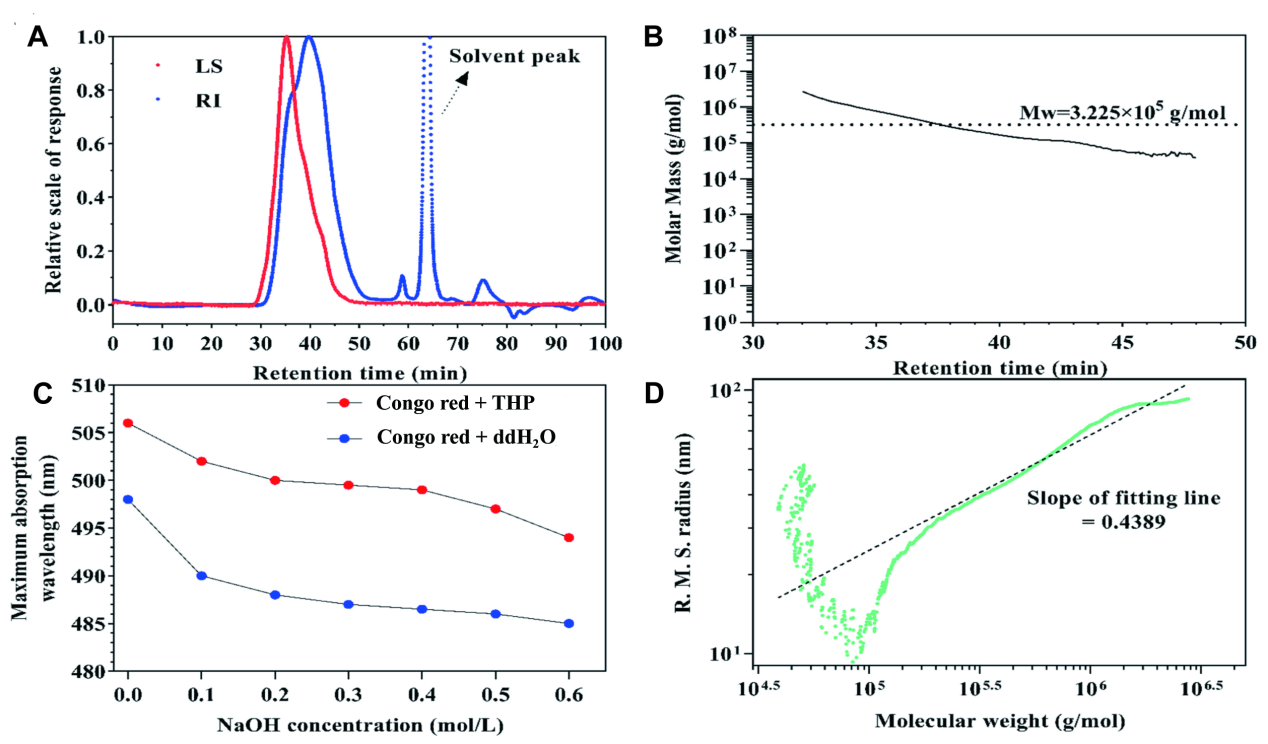


**Figure S3.** Homogeneity, molecular weight, and chain conformation. A) Light scattering (LS) and refractive index (RI) signals of THP. B) Molar mass and Mw of THP. C) Congo red assay. D) The plot of Rg versus molecular weight in a double logarithmic coordinate and the fitting line.


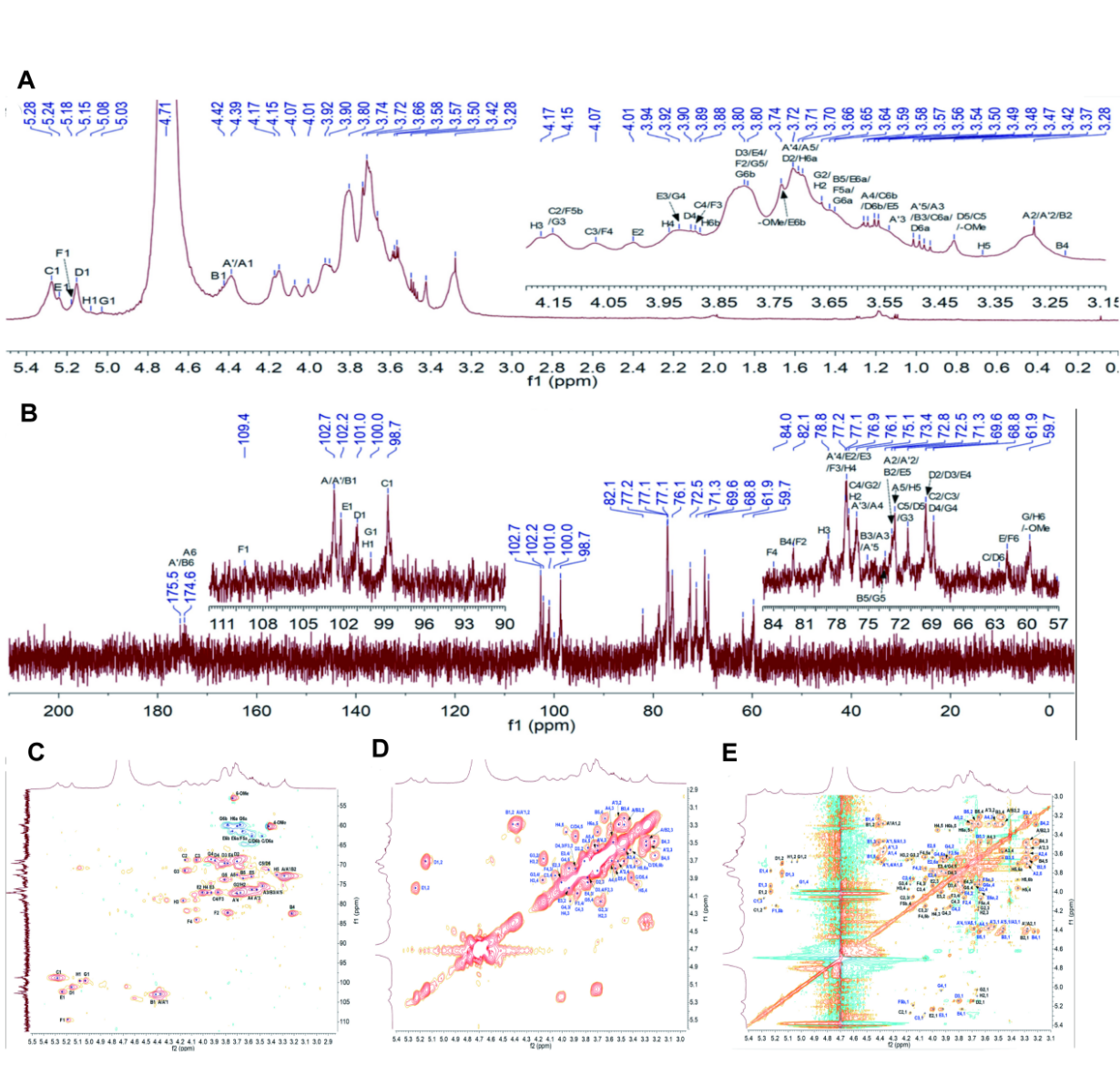


**Figure S4.** 1D and 2D NMR spectra of THP. A) ^1^H spectrum. B) ^13^C spectrum. C) HSQC. D) COSY. E) TOCSY. In the TOCSY spectrum, adjacent proton correlations were labeled in black; non-adjacent proton correlations were labeled in blue.


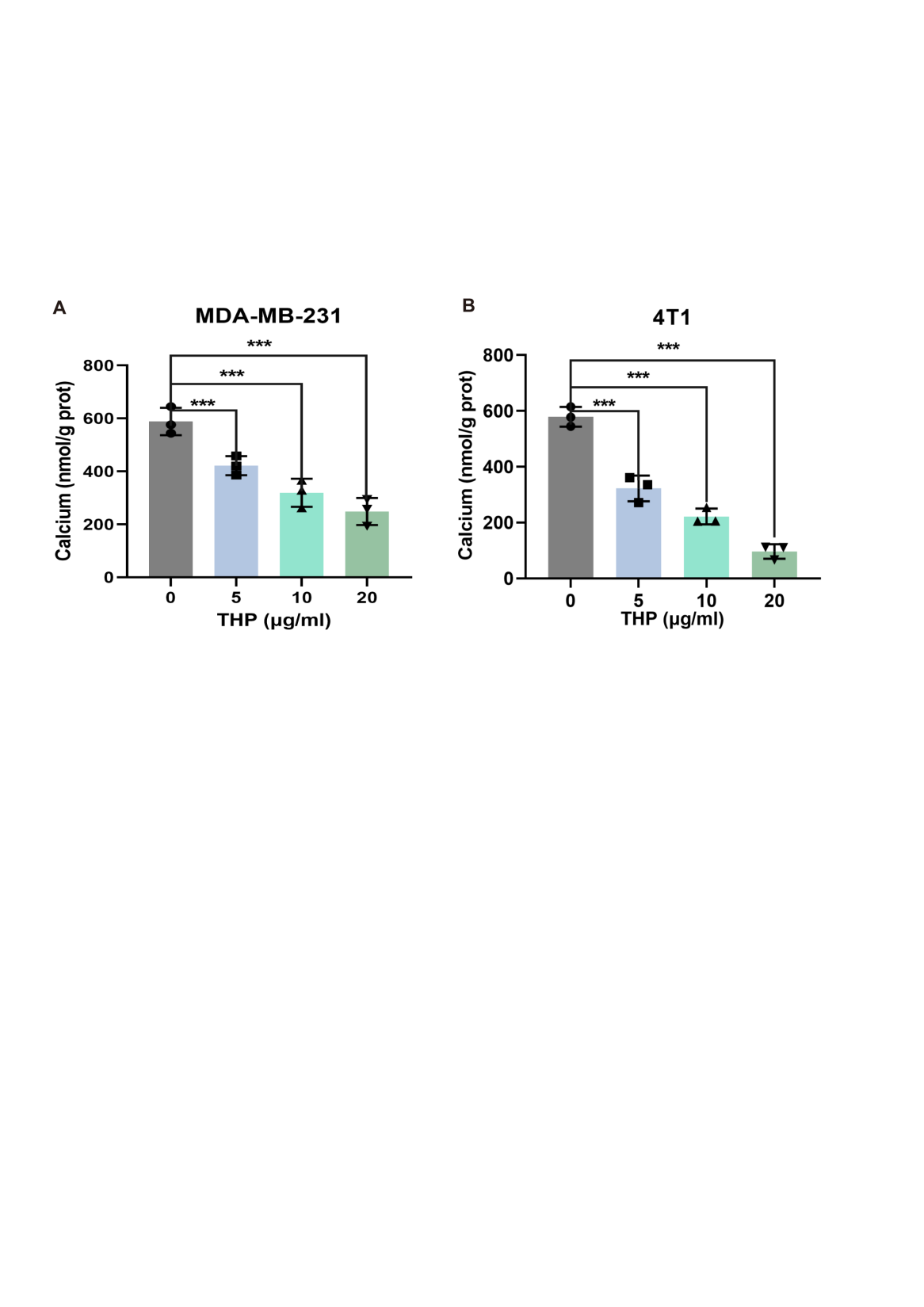


**Figure S5.**  THP depletes total cellular calcium content in a dose-dependent manner.

(A-B) MDA-MB-231 (A) and 4T1 (B) cells were treated with the indicated concentrations of THP for 24 h. Data represent mean ± SD. Significance was calculated using the one-way ANOVA with Dunnett’s multiple comparisons test. n= 3; ****p* < 0.001 vs. the control (0 μg/mL) group.

**
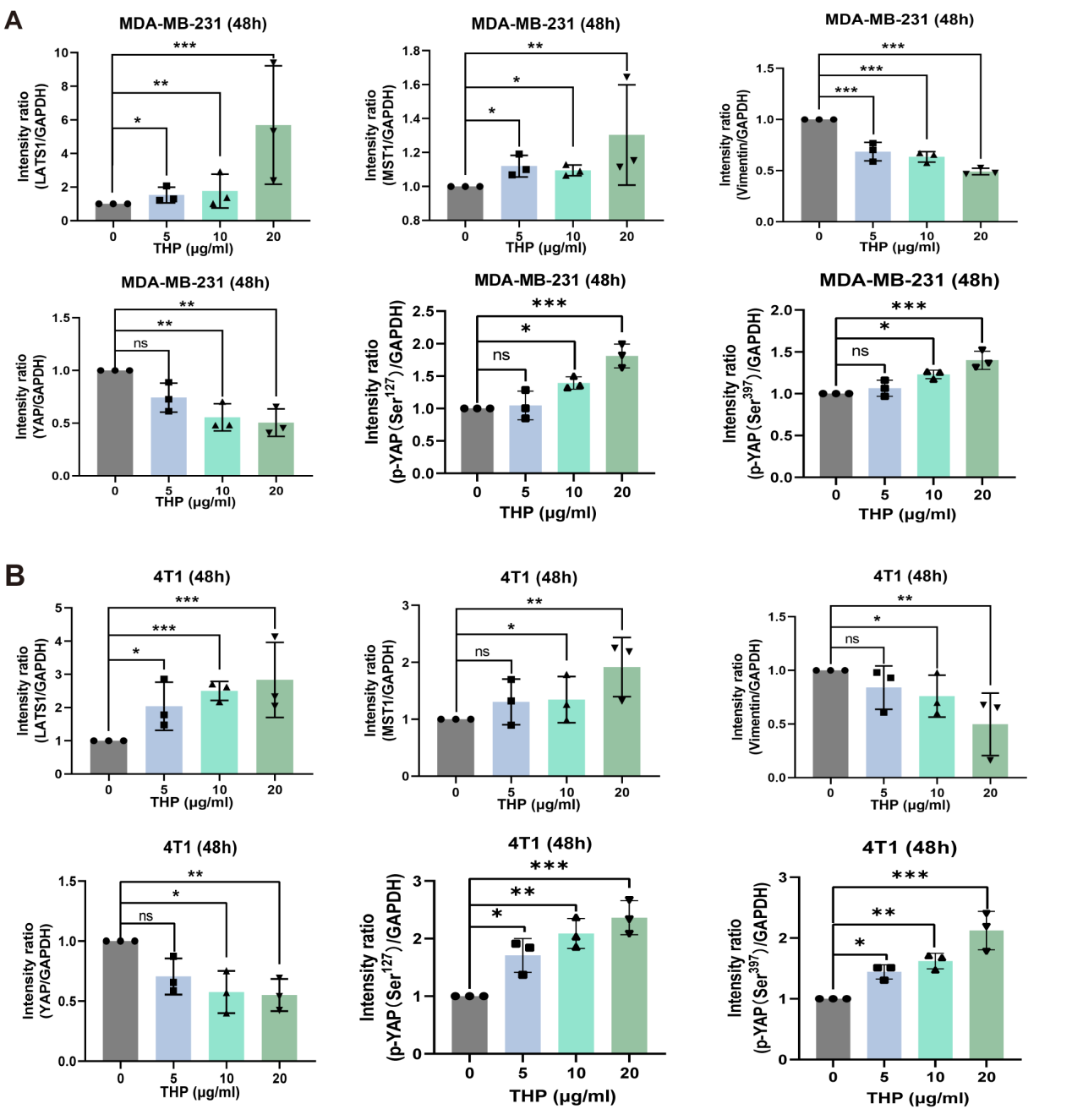
**

**Figure S6.** Quantification of the Western blot results from Figure 2C. A) Quantification of the Western blot results in MDA-MB-231 cells. B) Quantification of the Western blot results in 4T1 cells. Data represent mean ± SD. Significance was calculated using the one-way ANOVA with Dunnett’s multiple comparisons test. n= 3; **p* < 0.05; ***p* < 0.01; ****p* < 0.001.

**
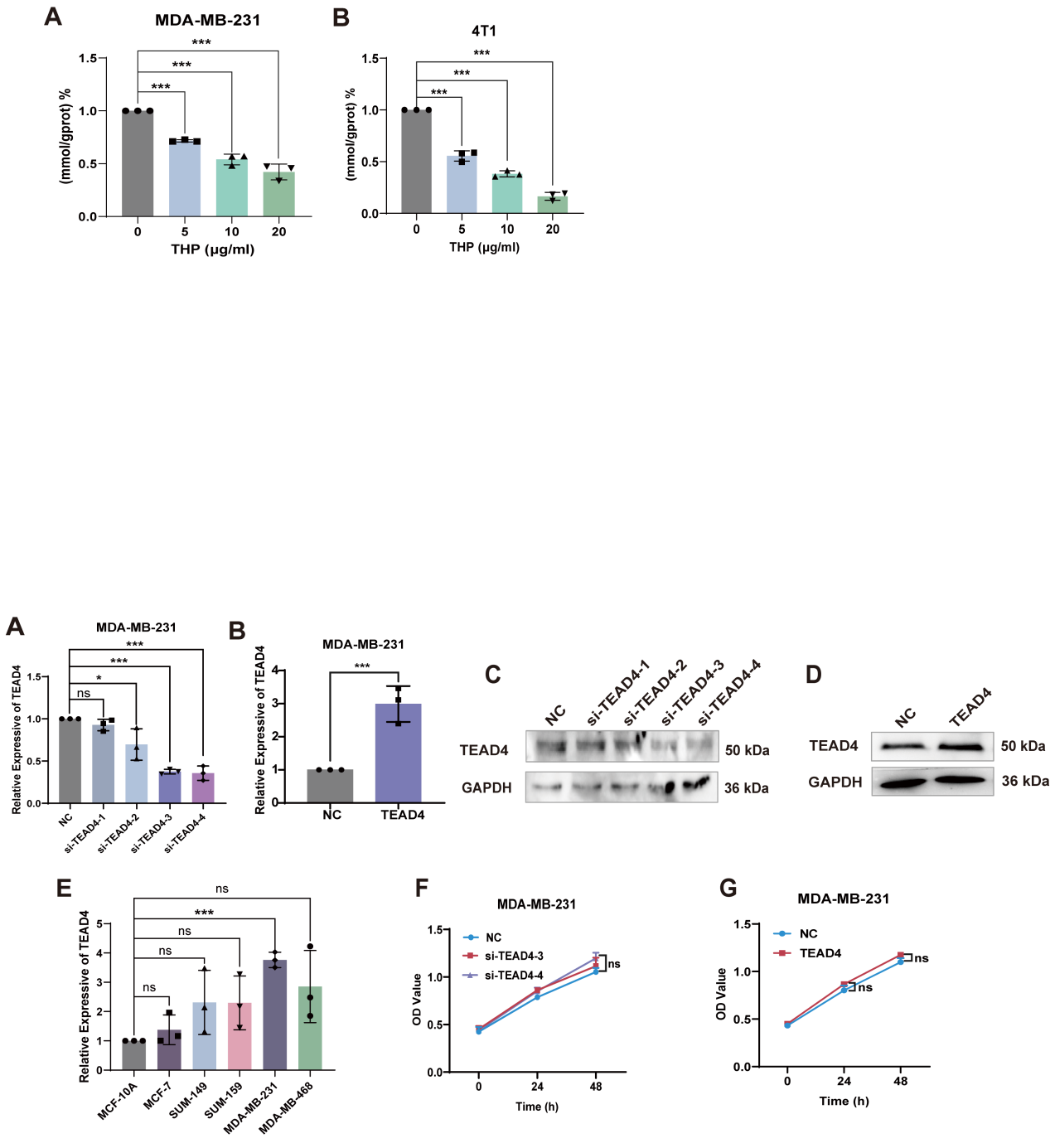
**

**Figure S7.** Construction of knockdown and overexpressing TEAD4 cell lines. A) Four plasmids with low TEAD4 knockdown were constructed and their mRNA expression was detected by RT-qPCR. B) TEAD4 overexpression plasmid was constructed and its mRNA expression was detected by RT-qPCR. C) Detection of protein level of TEAD4 knockdown effect of four plasmids. D) Detection of protein level of overexpressed TEAD4 plasmid. E) RT-qPCR quantified the relative mRNA expressions of TEAD4 in TNBC cell lines. F**–**G) Cell proliferation was detected after knockdown and overexpression of TEAD4. Data represent mean ± SD. Significance in (B) and (F**–**G) was calculated using the Unpaired T-test; Significance in (A) and (E) was calculated using the one-way ANOVA with Dunnett’s multiple comparisons test. n = 3; **p* < 0.05; ***p* < 0.01; ****p* < 0.001.


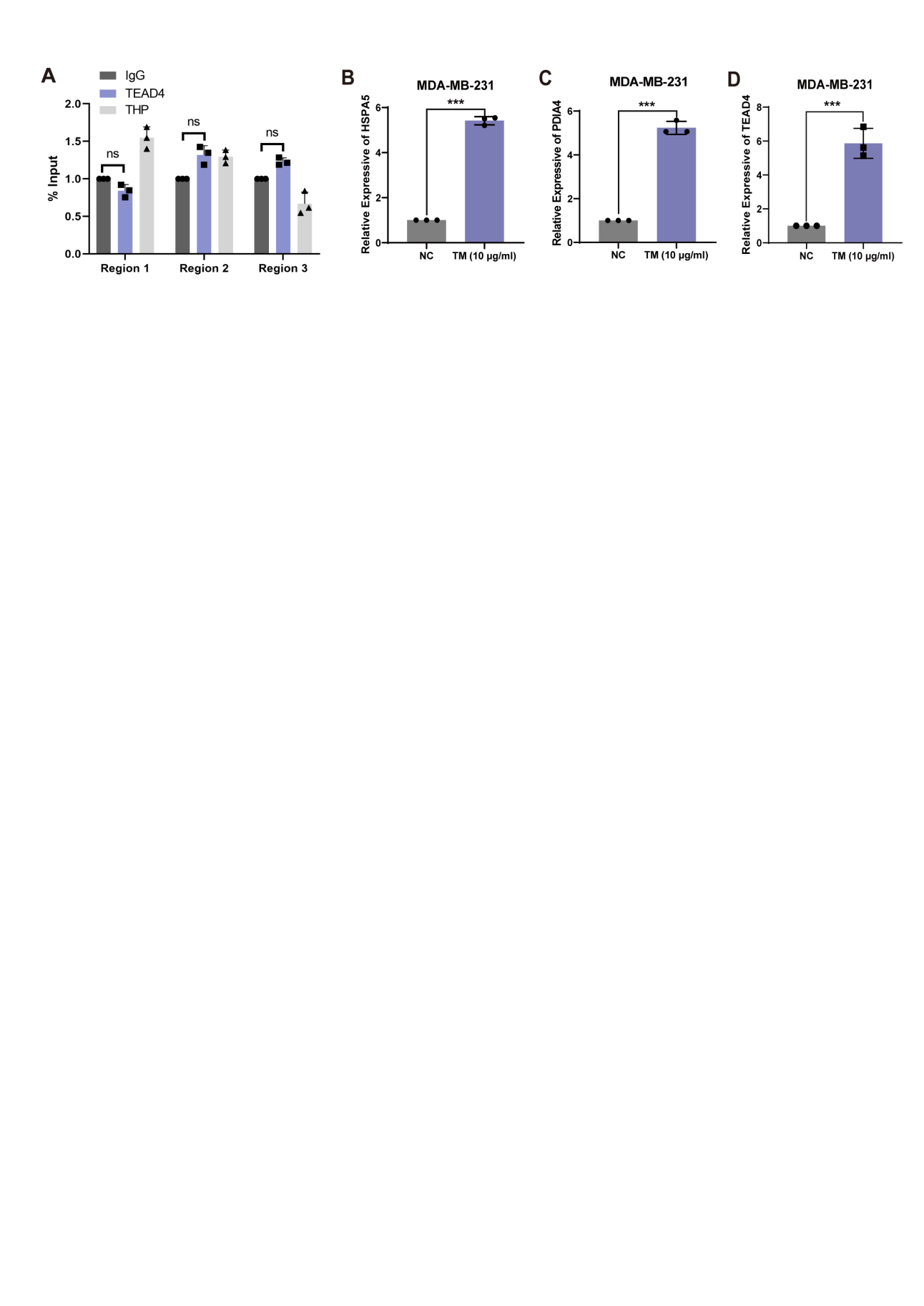


**Figure S8.** TEAD4 does not bind to the HSPA5 promoter, and ER stress upregulates HSPA5, PDIA4, and TEAD4. A) The ChIP-qPCR assay demonstrated that TEAD4 does not interact with any of the three predicted binding regions within the HSPA5 promoter in MDA-MB-231 cells. B**–**D) Under stress conditions, mRNA expression of HSPA5, PDIA4 and TEAD4 was detected by RT-qPCR. TM (Tunicamycin). Data represent mean ± SD, Significance in (A) was calculated using the one-way ANOVA with Dunnett’s multiple comparisons test; Significance in (B-D) was calculated using the Unpaired T-test; n = 3; ****p* < 0.001; ns, not significant.


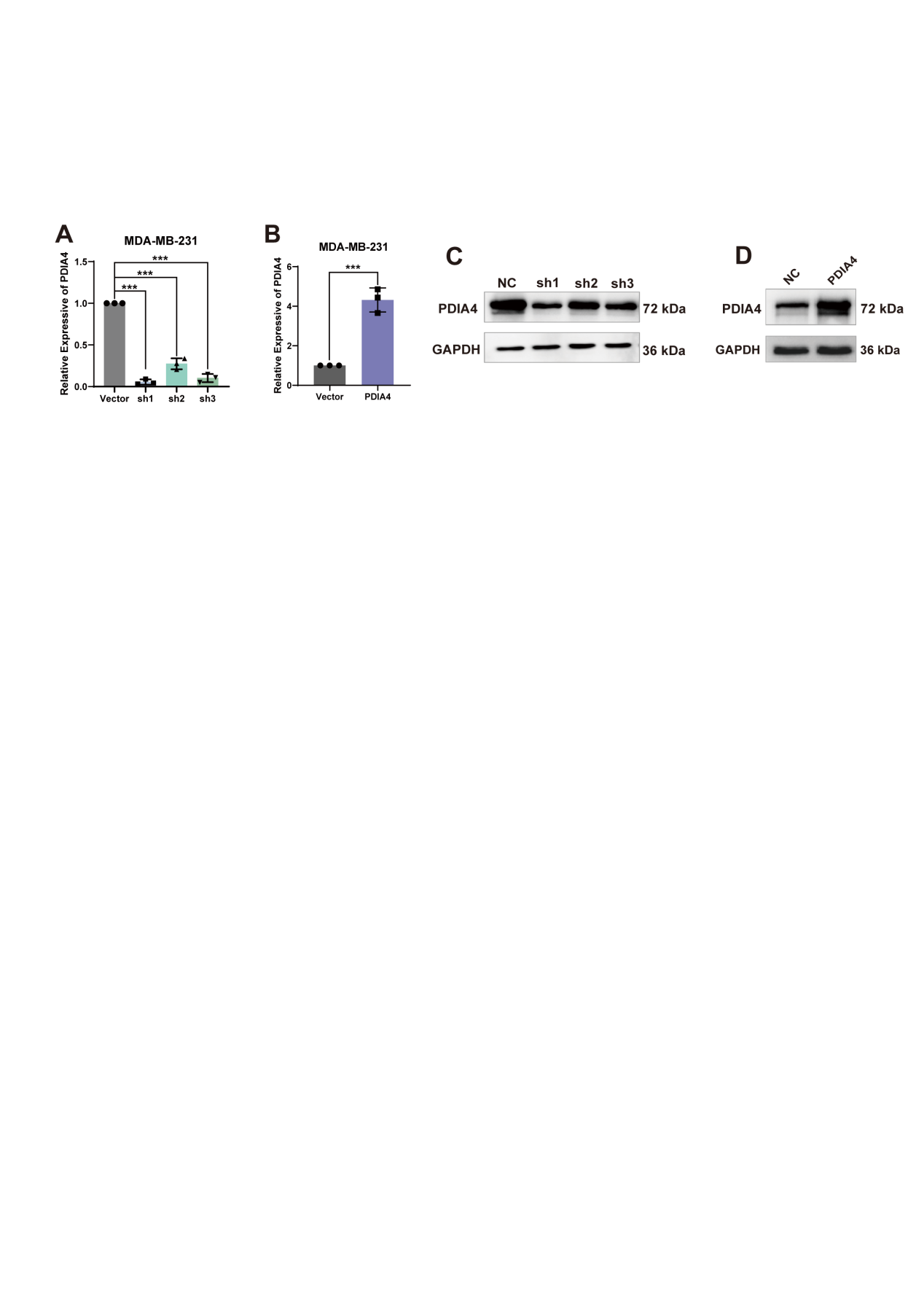


**Figure S9.** Molecular and protein validation of PDIA4 knockdown and overexpression in MDA-MB-231 cells. A) Three knockdown PDIA4 cell lines were constructed and their mRNA expression was detected by RT-qPCR. B) The mRNA expression in MDA-MB-231 cells after overexpression of PDIA4 was detected by RT-qPCR. C) The protein expression of four lentiviruses after PDIA4 knockdown was detected by WB. D) The protein expression level of PDIA4 after overexpression was detected by WB. Data represent mean ± SD, Significance in (A) was calculated using the one-way ANOVA with Dunnett’s multiple comparisons test; Significance in (B) was calculated using the Unpaired T-test. n = 3; ****p* < 0.001.


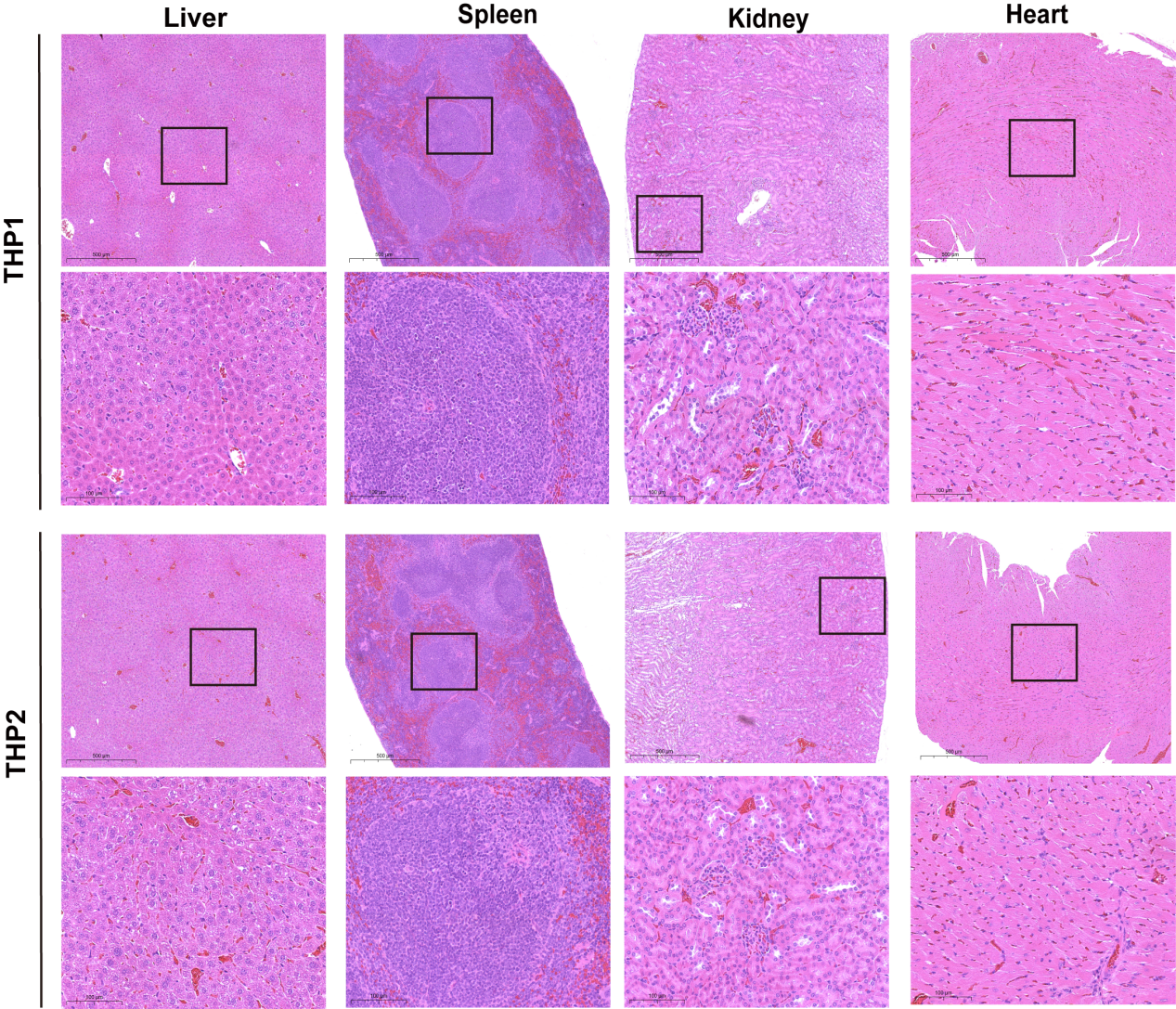


**Figure S10.** Representative HE-stained sections of the liver, kidney, heart, and spleen from THP-treated mice, confirming normal tissue architecture.


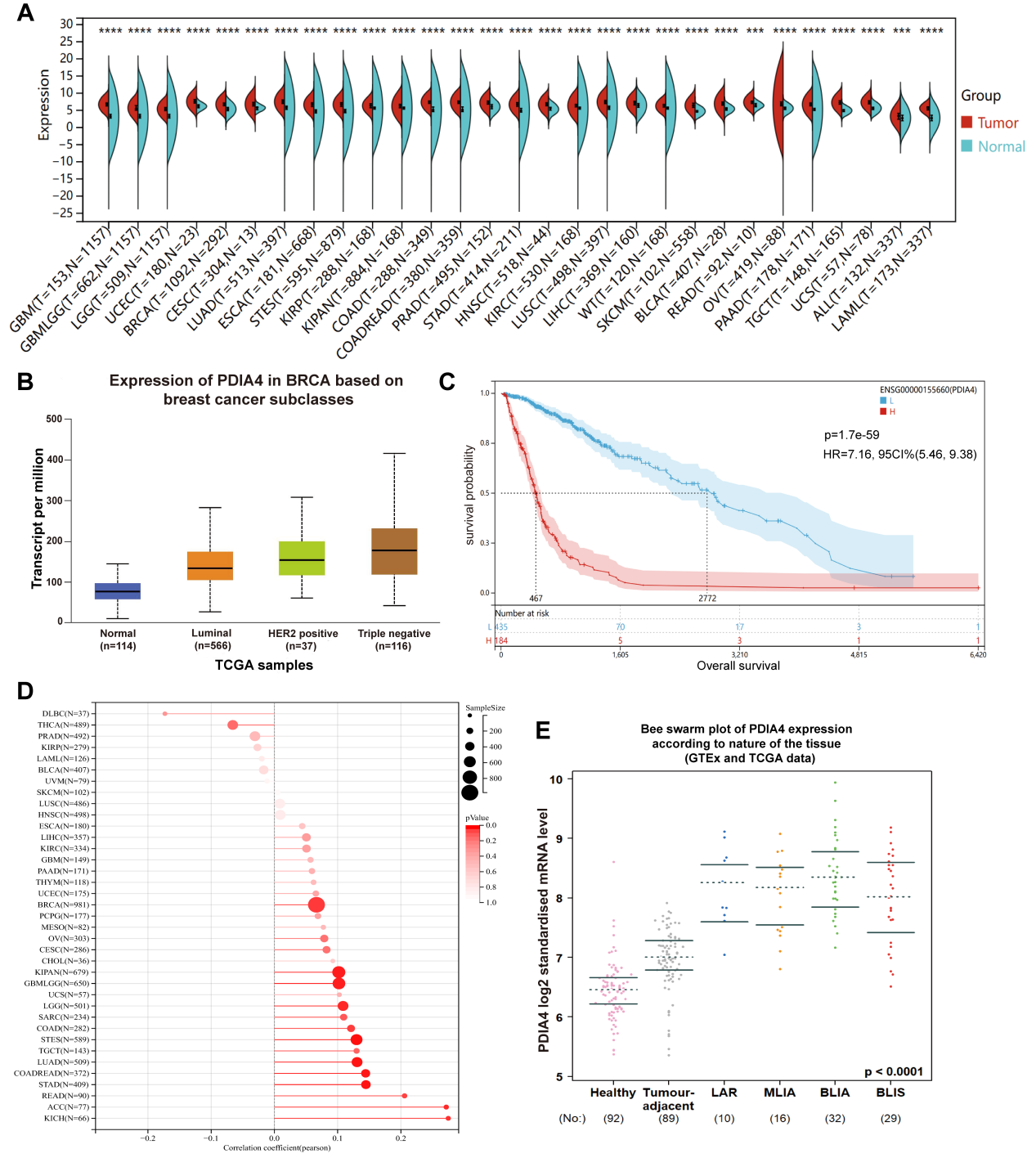


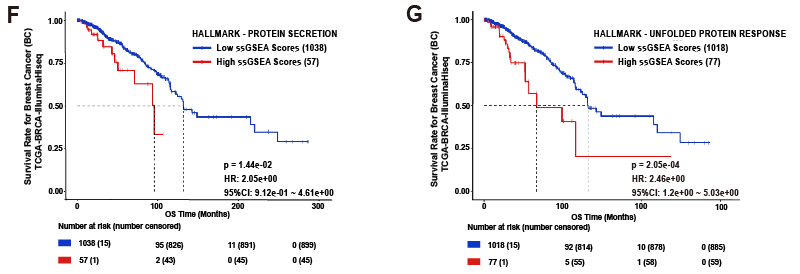


**Figure S11.** Pan-cancer multi-omics data resource and integrated analysis of PDIA4. A) Expression of PDIA4 in Pan-cancer. B) The expression level of PDIA4 in multiple subtypes of BRCA. C) Effect of PDIA4 on survival of BRCA. D) PDIA4 genomic heterogeneity and correlation with TMB. E) The mRNA expression levels of PDIA4 in normal tissues, tumor-adjacent tissues and multiple TNBC subtypes. Luminal Androgen Receptor (LAR), Mesenchymal-Like Immune-Activated (MLIA), Basal-Like Immune-Activated (BLIA), Basal-Like Immune-Suppressed (BLIS). F) Kaplan-Meier survival curves were utilized to analyze the OS of breast cancer patients stratified by different ssGSEA scores, aiming to explore the relationship between protein secretion level and OS time. A log- rank test was performed, and the result indicated a significant difference (*p* = 0.0144). G) Kaplan-Meier survival curves were used to analyze OS in breast cancer patients stratified by ssGSEA scores for the UPR signature. The association between UPR activity and OS was evaluated using log-rank test, revealing a statistically significant difference (*p* = 0.000205).

**Table S1.** Total carbohydrate, total uronic acid, protein content, and yield of THP

| Sample | Total carbohydrate | Total uronic acid | Protein | Yield |
| --- | --- | --- | --- | --- |
| THP | 83.5% | 24.73% | < 0.01% | 67.04% |

**Table S2.** Monosaccharide Composition of THP

| Monosaccharide | Retention times of standards (min) | Retention times of THP (min) | Composition of THP (molar ratio) |
| --- | --- | --- | --- |
| Fuc | 4.28 | ND | ND |
| Rha | 8.30 | 8.38 | **2.95** |
| Ara | 8.78 | 8.85 | **11.89** |
| Gal | 11.04 | 11.10 | **35.77** |
| Glc | 12.57 | 12.68 | **1.53** |
| Xyl | 14.93 | ND | ND |
| Man | 15.63 | 15.95 | **20.90** |
| Fru | 18.83 | ND | ND |
| Rib | 20.84 | ND | ND |
| GalA | 35.98 | ND | ND |
| GulA | 36.86 | ND | ND |
| GlcA | 39.06 | 38.45 | **26.95** |
| ManA | 41.90 | ND | ND |

ND = not detected.
